# Glycolysis regulates pollen tube polarity via Rho GTPase signaling

**DOI:** 10.1371/journal.pgen.1007373

**Published:** 2018-04-27

**Authors:** Wei Chen, Pingping Gong, Jingzhe Guo, Hui Li, Ruizi Li, Weiman Xing, Zhenbiao Yang, Yuefeng Guan

**Affiliations:** 1 Shanghai Center for Plant Stress Biology, Chinese Academy of Sciences, Shanghai, China; 2 FAFU-UCR Joint Center for Horticultural Biology and Metabolomics, Haixia Institute of Science and Technology, Fujian Agriculture and Forestry University, Fuzhou, Fujian, China; 3 University of Chinese Academy of Sciences, Shanghai, China; 4 Center for Plant Cell Biology, Institute of Integrated Genome Biology, and Department of Botany and Plant Sciences, University of California, Riverside, California, United States of America; University of Arizona, UNITED STATES

## Abstract

As a universal energy generation pathway utilizing carbon metabolism, glycolysis plays an important housekeeping role in all organisms. Pollen tubes expand rapidly via a mechanism of polarized growth, known as tip growth, to deliver sperm for fertilization. Here, we report a novel and surprising role of glycolysis in the regulation of growth polarity in *Arabidopsis* pollen tubes via impingement of Rho GTPase-dependent signaling. We identified a *cytosolic phosphoglycerate kinase* (*pgkc-1*) mutant with accelerated pollen germination and compromised pollen tube growth polarity. *pgkc-1* mutation greatly diminished apical exocytic vesicular distribution of REN1 RopGAP (Rop GTPase activating protein), leading to ROP1 hyper-activation at the apical plasma membrane. Consequently, *pgkc-1* pollen tubes contained higher amounts of exocytic vesicles and actin microfilaments in the apical region, and showed reduced sensitivity to Brefeldin A and Latrunculin B, respectively. While inhibition of mitochondrial respiration could not explain the *pgkc-1* phenotype, the glycolytic activity is indeed required for PGKc function in pollen tubes. Moreover, the *pgkc-1* pollen tube phenotype was mimicked by the inhibition of another glycolytic enzyme. These findings highlight an unconventional regulatory function for a housekeeping metabolic pathway in the spatial control of a fundamental cellular process.

## Author summary

Glycolysis, which breaks down glucose to produce energy, has long been considered a “housekeeping” pathway in living cells, i.e., it helps maintain basic cellular functions. Here, we found that the glycolysis pathway plays an unconventional regulatory role in cell polarity, i.e., the intrinsic asymmetry in the shape, structure, and organization of cellular components. Mutation in the gene encoding the glycolytic enzyme cytosolic phosphoglycerate kinase (PGKc) leads to swollen and shorter pollen tubes in *Arabidopsis thaliana*, which is associated with the over-activation of Rho GTPase—a master regulator of cell polarity. Our results suggest that this phenomenon is caused by a specific regulatory role of cytosolic glycolysis rather than the global energy supply or moonlighting functions of glycolytic enzymes that modulate pollen tube growth polarity. Our findings shed light on the diverse biological roles of glycolysis in plants beyond simple “housekeeping” functions.

## Introduction

Glycolysis, which generates two ATP from each glucose molecule and produces two pyruvate molecules to fuel the mitochondrial tricarboxylic acid cycle, is a central enzymatic process in carbon metabolism. In addition, glycolysis also produces metabolic intermediates and reduced cofactors for secondary metabolism, as well as amino acid and fatty acid biosynthesis [1, 2]. Recent studies have hinted at a role for energy in the regulation of cellular processes independent of the housekeeping function. For instance, aldolase, a glycolytic enzyme, acts as a sensor of glucose availability in mammalian cells, and represses the energy sensing AMP-dependent kinase (AMPK) pathway, which is known to coordinate cell growth, metabolism, and cell polarity [3–6]. Therefore, glycolysis may play a regulatory role in determining cell polarity regulation, although direct evidence for this role is lacking thus far.

Polarized cell growth is a conserved cellular process shared by many diverse systems in eukaryotic species, such as the mating tubes in budding yeast, cell growth and morphogenesis in fission yeast and filamentous fungi, axon outgrowth in animals, and root hair and pollen tube formation in plants. Pollen tubes are a well-established and favorite model system for studying cell polarity formation and polar cell growth [7]. Pollen tubes are among the most rapidly extending polarized cells, growing at rates of up to 250 nm per second [8]. The rapid tip growth exhibited by pollen tubes is supported by cytoskeletal organization/dynamics and vesicular trafficking coordinated by a conserved signaling network dependent upon a plant Rho GTPase (ROP1) [7–12]. ROP1 is activated in the apical region, where it orchestrates F-actin dynamics and calcium homeostasis to dynamically maintain apical growth in the pollen tube [11, 13]. REN1, a RhoGAP, acts as a global inhibitor to spatially restrict ROP1 activity to the apical plasma membrane at the pollen-tube tip region [14]. This self-organizing ROP signaling network is comprised of multiple coordinated pathways and feedback loops, providing a robust molecular linkage between the cytoskeleton, vesicular trafficking, and polarity formation [11–13, 15–19].

It is conceivable that the rapid tip growth exhibited by pollen tubes is extremely energy-demanding. Overall elevations in energy metabolism in pollen tubes appears to rely on plastid-localized glycolysis and mitochondrial-localized respiration pathways [20–23]. As a result, respiration rates in pollen tubes are up to ten times greater than those in vegetative tissues [24]. In addition, the ethanol fermentation pathway is also active in support of pollen tube growth [25]. Apart from an overall increase in energy metabolism, rapid tip growth may also require a tight spatiotemporal regulation of energy production, given the tightly regulated spatiotemporal dynamic of the aforementioned processes.

Phosphoglycerate kinase (PGK) is a key enzyme in the glycolytic pathway, responsible for catalyzing the reversible conversion of 1,3-biphosphoglycerate (1,3BPG) to 3-phosphoglycerate (3PG). Here, we report that the Arabidopsis cytosolic phosphoglycerate kinase (PGKc) plays a regulatory role in the regulation of pollen tube polarity by modulating the apical distribution of the REN1 RhoGAP, and thus the activity of the apical ROP1 RhoGTPase as well. This action of PGKc is specific for the cytosolic glycolysis pathway and is independent of mitochondrial respiration. Our findings provide the first conclusive evidence that glycolysis plays an important and specific role in the regulation of cell polarity.

## Results

### PGKc mutations resulted in pollen tube growth depolarization and accelerated pollen germination

**Fig 1:**
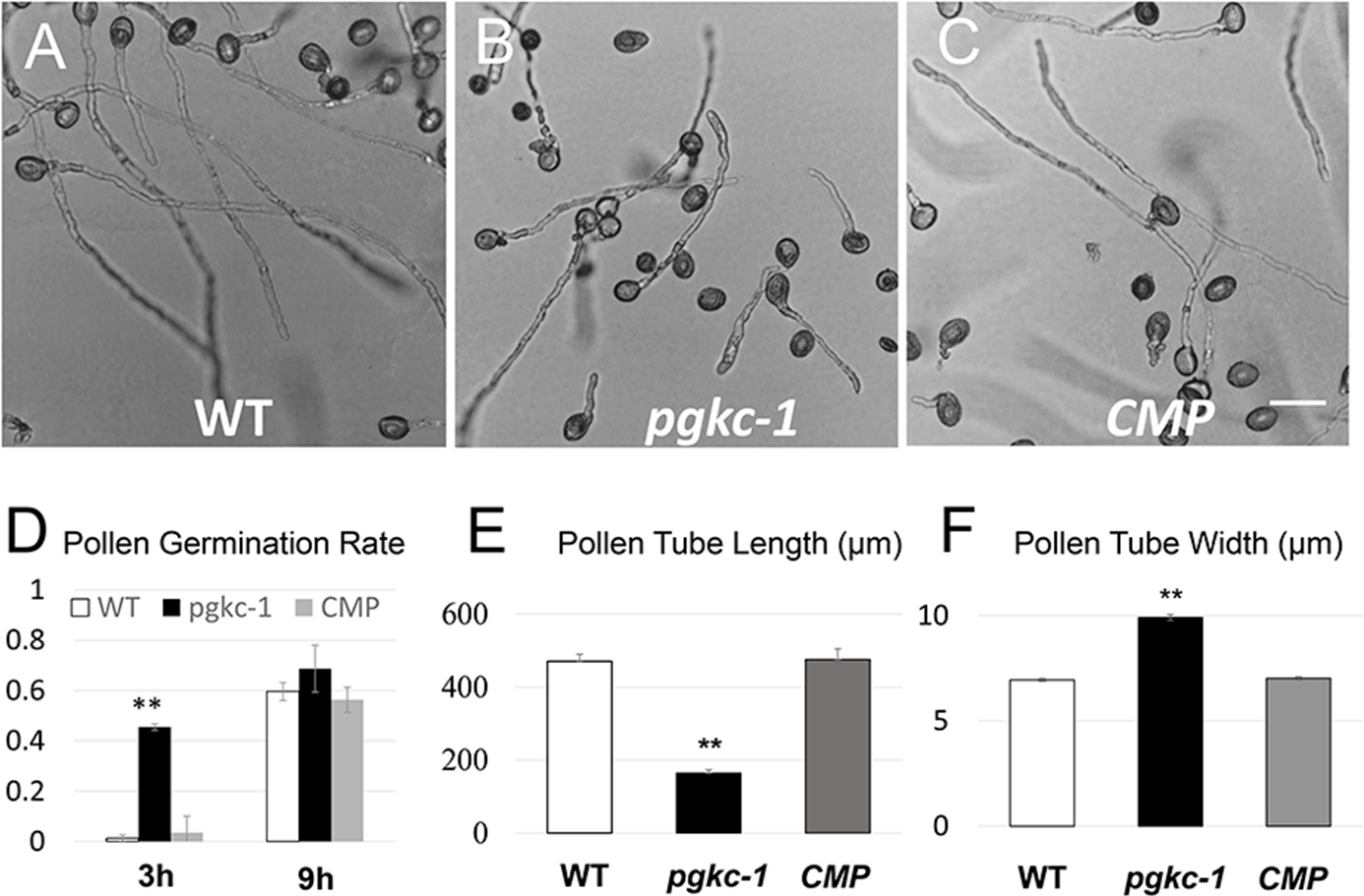
*pgkc-1* mutant exhibits enhanced pollen germination and growth depolarization. **(A)** WT pollen tube morphology. **(B)** *pgkc-1* (SALK_066422C) pollen tube morphology. **(C)** Complemented *pgkc-1* pollen tube morphology. Scale bar = 50 μm. **(D)** Pollen germination rate at 3 h and 9 h, respectively. *pgkc-1* germinated at higher rates than WT and complemented pollen, especially at the early time point. (**E**) Pollen tube length of WT, *pgkc-1* mutant, and genetically complemented *pgkc-1* plants at 9 h after germination. **(F)** Pollen tube width of WT, *pgkc-1* mutant, and genetically complemented *pgkc-1* plants at 9 h after germination. Bars represent mean ± SEM. Asterisks indicate significant differences (** = p < 0.001) versus WT as determined by Student’s *t*-test.

To discover new genes regulating pollen tube polarity, we performed a genetic screen for *Arabidopsis thaliana* mutants from the SALK collection of individually indexed homozygous T-DNA insertion lines presenting altered growth polarity. Among over 8000 individual lines screened, pollen tubes from SALK_066422C were identified to present defective polarized growth in *in vitro* germination medium. According to the annotation, SALK_066422C contains a T-DNA inserted into the 5^th^ exon of AT1G79550, which encodes a cytosolic phosphoglycerate kinase (designated hereafter as PGKc) (**S1A and S1B Fig**). We therefore designated the mutant as *pgkc-1*. The *pgkc-1* mutant pollen germinated at a much faster rate than wild type (WT) plants (**Fig 1D**). However, *pgkc-1* mutant pollen tubes were significantly shorter than WT ones after 9 h (**Fig 1A, 1B and 1E**). Moreover, the majority of mutant pollen tubes were swollen relative to WT, exhibiting irregular morphology and wider tube width (**Fig 1A, 1B and 1F**).

We also obtained an independent allele mutant with a T-DNA insertion in the 3^rd^ intron of *PGKc* (SALK_062377, designated *pgkc-2*), which showed similar pollen tube phenotypes (**S1A, S1B and S1G Fig**). Quantitative reverse transcription polymerase chain reaction (Q-RT-PCR) showed that both *pgkc-1* and *pgkc-2* are knockout mutants for *PGKc* (**S1C Fig**). We also performed a backcross of *pgkc-1* with WT plants, where F2 progeny *pgkc-1* homozygous plants showed defects in pollen tube polarity while WT progeny remained normal (**S1E and S1F Fig**). This indicates the co-segregation of the *pgkc-1* locus with the mutant phenotype. Finally, the *pgkc-1* mutant was rescued by introducing *PGKc* genomic sequences, including the native promoter and terminator (**Fig 1C to 1F and S1D Fig**). Taken together, our results confirm that loss of *PGKc* is indeed responsible for the pollen tube polarity phenotype. The vegetative growth and flowering of *pgkc-1* plants were slightly delayed relative to WT, but mutant plant morphology was normal otherwise (**S2A–S2C Fig**).

The *Arabidopsis* genome contains three *PGK* genes, AT1G79550 (PGKc), AT3G12780, and AT1G56190. Recent reports have shown that AT1G79550 encodes the sole cytosolic PGK, while AT1G79550 and AT3G12780 are plastid localized [26]. We also performed subcellular localization analysis using a GFP fusion protein. Consistent with the results of a previous study, we found PGKc to be localized to the cytoplasm and nuclei while the other 2 PGKs were localized to the chloroplasts (plastids) (**S3A–S3F Fig**). Finally, both a previous study and publicly available microarray expression data showed that PGKc is expressed ubiquitously in most plant tissues, including pollen (https://genevestigator.com/) [26].

### Alteration of actin microfilaments and vesicular trafficking in *pgkc-1* pollen tubes

**Fig 2:**
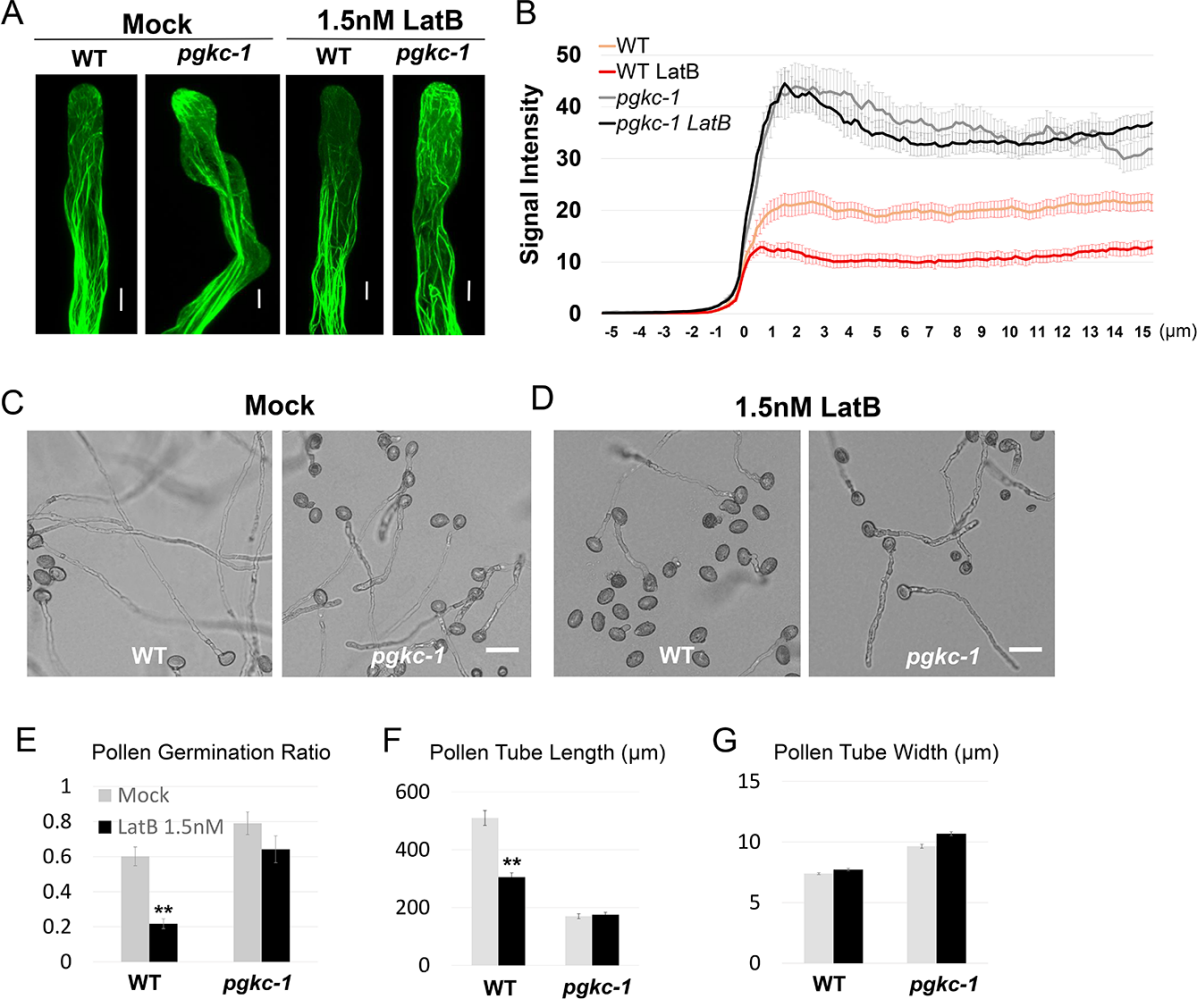
F-actin dynamics in the *pgkc-1* mutant. **(A)** Lifeact-mEGFP signal in WT and *pgkc-1* pollen tubes with mock or 1.5 nM LatB treatment. Scale bar = 5 μm. **(B)** Average GFP signal intensity along WT and *pgkc-1* pollen tubes with mock or 1.5 nM LatB treatment. Measurements were performed as described in Materials and Methods. Thirty-five pollen tubes were measured for each sample. The 0 μm indicates the position of the extreme tip. Orange line indicates WT pollen tube; red line indicates WT pollen tube treated with 1.5 nM LatB; gray line indicates *pgkc-1* pollen tube; black line indicates *pgkc-1* pollen tube treated with 1.5 nM LatB. Error bars on curves indicate standard error of the mean. **(C and D)** WT and *pgkc-1* pollen tube growth when subjected to mock medium **(C)** or 1.5 nM LatB **(D)** treatment. Scale bar = 50 μm. **(E to G)** WT and *pgkc-1* plant pollen germination **(E)**, pollen tube length **(F)**, and pollen tube width **(G)** when subjected to mock or 1.5 nM LatB treatment. Bars represent mean ± SEM. Asterisks indicate significant differences versus mock treatment as determined using Student’s *t*-test (** = p < 0.001).

Our surprising findings regarding *PGKc* knockouts prompted us to assess how a housekeeping glycolytic enzyme can regulate cell polarity. We first performed a series of assays to assess *pgkc-1* mutant phenotype cellular mechanisms with known links to cell polarity defects. The spatiotemporal dynamics of apical actin microfilaments (F-actin) and vesicle trafficking is crucial for generation of cell polarity and pollen tube tip growth [7, 13]. We observed F-actin organization in *pgkc-1* pollen tubes by introducing a Lifeact-mEGFP marker via crossing [2, 27]. In WT pollen tubes, highly dynamic fine F-actin structures were observed in the apical region, dense F-actin fringe structures were present in sub-apical regions, and parallel longitudinal F-actin bundles were found in shank regions (**Fig 2A**). Dynamic apical F-actin has been shown to be disrupted by treatment with 1.5 nM Latrunculin B (LatB), a chemical promoting actin depolymerization [9] [28] (**Fig 2A and 2B**). In *pgkc-1* pollen tubes, no significant difference was detected in the shank and sub-apical regions. However, fine F-actin filaments were significantly over-accumulated towards the apex of the apical tip region in *pgkc-1* pollen tubes, even after LatB treatment (**Fig 2A and 2B**). Indeed, treatment with 1.5 nM LatB had no significant effect on the germination, length, and morphology of *pgkc-1* mutant pollen tubes, but greatly inhibited similar mechanisms in WT pollen tubes (**Fig 2C to 2G**).Taken together, these results indicate that *pgkc-1* mutation promotes the accumulation of F-actin in the apical tip region of the pollen tube.

**Fig 3:**
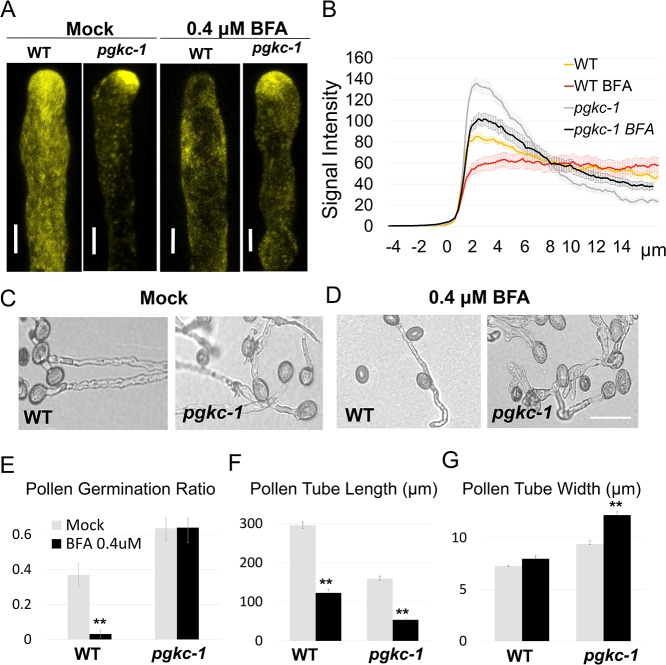
Vesicle trafficking in the *pgkc-1* mutant. **(A)** EYFP-RABA4D signal in WT and *pgkc-1* pollen tubes subjected to mock or 0.4 μM BFA treatment. Scale bar = 5 μm. **(B)** EYFP-RABA4D signal intensity. Measurements were performed as described in Materials and Methods. Fifteen to twenty pollen tubes from each sample were measured. 0 μm indicates the position of apical tip. Error bars on curves indicate standard error. **(C and D)** WT and *pgkc-1* pollen tube morphology when subjected to **(C)** mock and **(D)** 0.4 μM BFA treatment. Scale bar = 50 μm. **(E to G)** WT and *pgkc-1* plant pollen germination **(E)**, pollen tube length **(F)**, and pollen tube width **(G)** when subjected to mock or 0.4 μM BFA treatment. Bars represent mean ± SEM. Asterisks indicate significant differences versus mock treatment as determined using Student’s *t*-test (** = p < 0.001).

A previous study has shown that an increased level of apical F-actin leads to greater apical accumulation of exocytic vesicles [11]. Thus, we examined the distribution of exocytic vesicles in *pgkc-1* mutant pollen tubes. The Rab GTPase RABA4D is a pollen-specific *Arabidopsis* homolog of animal Rab11 known to localize to post-Golgi compartments, including exocytic vesicles in pollen tube tips [14, 29]. We introduced an EYFP-RABA4D marker into *pgkc-1* pollen tubes via crossing. In WT pollen tubes, EYFP-RABA4D-labeled membrane compartments were punctuated and enriched in the tip region (**Fig 3A**). In *pgkc-1* pollen tubes, the apical distribution pattern of RABA4D was similar to that of WT pollen tubes (**Fig 3A**), but quantification of EYFP-RABA4D signal showed that apical EYFP-RABA4D compartments were much more enriched in *pgkc-1* pollen tube compared to WT despite lower signal intensity in the shank region (**Fig 3B**), a similar pattern to that observed in pollen tubes with ROP1 over-activation [11].

We next examined whether *pgkc-1* pollen tubes respond differently to Brefeldin A (BFA), an inhibitor which interrupts vesicle trafficking by inhibiting vesicle formation from TGN and recycling endosomes [30–33]. Application of 0.4 μM BFA abolished the apical enrichment of RABA4D signal observed in WT pollen tubes, but had a markedly reduced effect on RABA4D localization in *pgkc-1* pollen tubes (**Fig 3A and 3B**). Moreover, BFA greatly inhibited WT pollen germination but only moderately affected *pgkc-1* pollen germination (**Fig 3C to 3G**). Interestingly, *pgkc-1* pollen tubes exhibited enhanced growth depolarization when treated with BFA (**Fig 3C to 3G**). Germinated *pgkc-1* pollen tubes were shorter and wider, and multiple tips occasionally formed from a single pollen grain (**Fig 3D**). These results suggested that the *pgkc-1* mutation appears to enhance the production or accumulation of exocytic vesicles in pollen grains and tubes. This altered vesicular trafficking behavior in pollen tube tips is consistent with the aforementioned observed over-accumulation of apical F-actin [11].

### *pgkc-1* mutation affects REN1 distribution at the pollen tube apex

**Fig 4:**
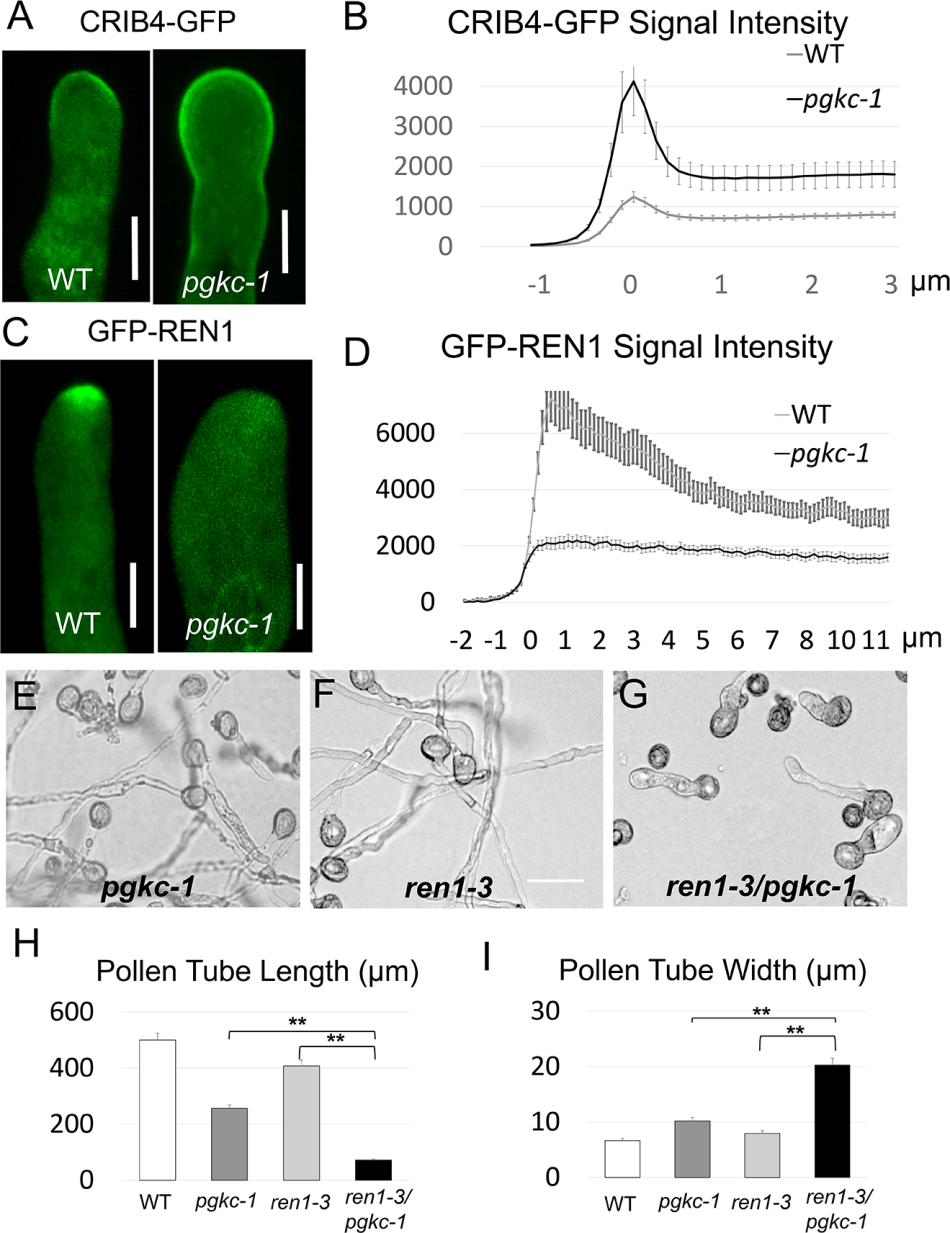
ROP1 signaling in the *pgkc-1* mutant. **(A)** Active ROP1 visualized by CRIB4-GFP signal in WT and *pgkc-1* pollen tubes. Scale bar = 5μm. **(B)** Average CRIB4-GFP signal intensity along WT and *pgkc-1* pollen tubes. **(C)** GFP-REN1 localization in WT and *pgkc-1* pollen tubes. Scale bar = 5 μm. **(D)** Average GFP-REN1 signal intensity along WT and *pgkc-1* pollen tubes. Measurements were performed as described in Materials and Methods. Fifteen pollen tubes from each sample were measured. The 0 μm label indicates the position of the apical tip. Error bars on curves indicate standard error of the mean. **(E to G)** Pollen tube morphology of *pgkc-1* **(E)**, *ren1-3* **(F)**, and *ren1-3/pgkc-1* double mutant plants **(G)**. Scale bar = 50 μm. **(H and I)** Pollen tube length **(H)** and width **I)** of WT, *pgkc-1*, *ren1-3*, and *ren1-3/pgkc-1* double mutant plants. Bars represent mean ± SEM. Asterisks indicate significant differences versus single mutant plant as determined using Student’s *t*-test (** = p < 0.001).

Given the role of ROP1 GTPase signaling in regulating F-actin dynamics and vesicle trafficking [7, 11, 13], we speculated that the F-actin dynamics and vesicle trafficking phenotype in the *pgkc-1* mutant may be linked to altered ROP1 signaling. RIC4 binds active ROP1 via its CRIB4 domain. CRIB4-GFP localization to the plasma membrane indicates ROP1 activity in pollen tubes [14]. To evaluate whether ROP1 activity was altered in *pgkc-1* mutants, we introduced CRIB4-GFP into the *pgkc-1* mutant background. CRIB4-GFP localization to the apical plasma membrane was significantly broader in *pgkc-1* mutant pollen tubes than in WT (**Fig 4A**). Quantification revealed stronger CRIB4-GFP signal in *pgkc-1* pollen tubes than in WT counterparts (**Fig 4B**). This result suggested that active ROP1 levels were indeed excessive in *pgkc-1* pollen tubes.

A previous study has shown that the RhoGAP REN1 is an important regulator of ROP1 negative feedback loops. REN1 is localized to exocytic vesicles in the pollen tube tip [14]. A mutation in *REN1* causes swollen pollen tubes and is correlated with hyper-activation of ROP1 [14]. We therefore introduced a GFP-REN1 reporter into *pgkc-1* plants to observe the subcellular distribution of this negative feedback regulator of ROP signaling. Consistent with the previous study, GFP-REN1 was enriched in the apical region in an inverted-cone pattern, reminiscent of the distribution of RABA4D-labeled vesicles (**Figs 4C and 3A**). Strikingly, in *pgkc-1* pollen tubes, this apical localization of GFP-REN1 was abolished in stark contrast to the enhanced apical accumulation of RABA4D-labeled vesicles observed (**Fig 4C and 4D**). These results indicate that *pgkc-1* mutation disrupted apical localization of REN1, which may be associated with ROP1 hyper-activation.

To examine the functional interaction between PGKc and REN1, we generated double mutants using *pgkc-1* and *ren1-3* mutant plants. *ren1-3* plants contain a weak mutation consisting of a C terminus truncation which confers a mild polarization defect [14] **(S4 Fig**). If PGKc functionally interacts with REN1, the tip-targeting defect of REN1 present in *pgkc-1* plants would have a synergistic effect with the phenotype observed in the *ren1-3* mutant. We found that in standard medium, *ren1-3* pollen tubes displayed near normal growth and morphology, while *pgkc-1* pollen tubes exhibited reduced growth and moderate polarity defects (**Fig 4E and 4F**). However, the pollen tubes of the *pgkc-1/ren1-3* double mutant plants were much shorter and dramatically more swollen compared to either single mutant (**Fig 4G to 4I**). These results indicate that a moderate REN1 defect in *ren1-3* was synergistically enhanced by *pgkc-1*, demonstrating the genetic interaction between PGKc and REN1.

### Glycolysis but not mitochondria respiration is responsible for *pgkc-1* pollen tube phenotype

We reasoned that PGKc, as a glycolytic enzyme, regulated pollen tube polarity through one or more of the following possible mechanisms: (1) pollen tube polarity may be linked to overall cellular ATP level, which is dependent on both glycolysis and mitochondrial respiration; (2) glycolysis may play a regulatory role in determining pollen tube polarity; and (3) PGKc may have evolved a new, so-called “moonlighting” function distinct from its role in glycolysis. We performed a series of assays to examine these possibilities.

**Fig 5:**
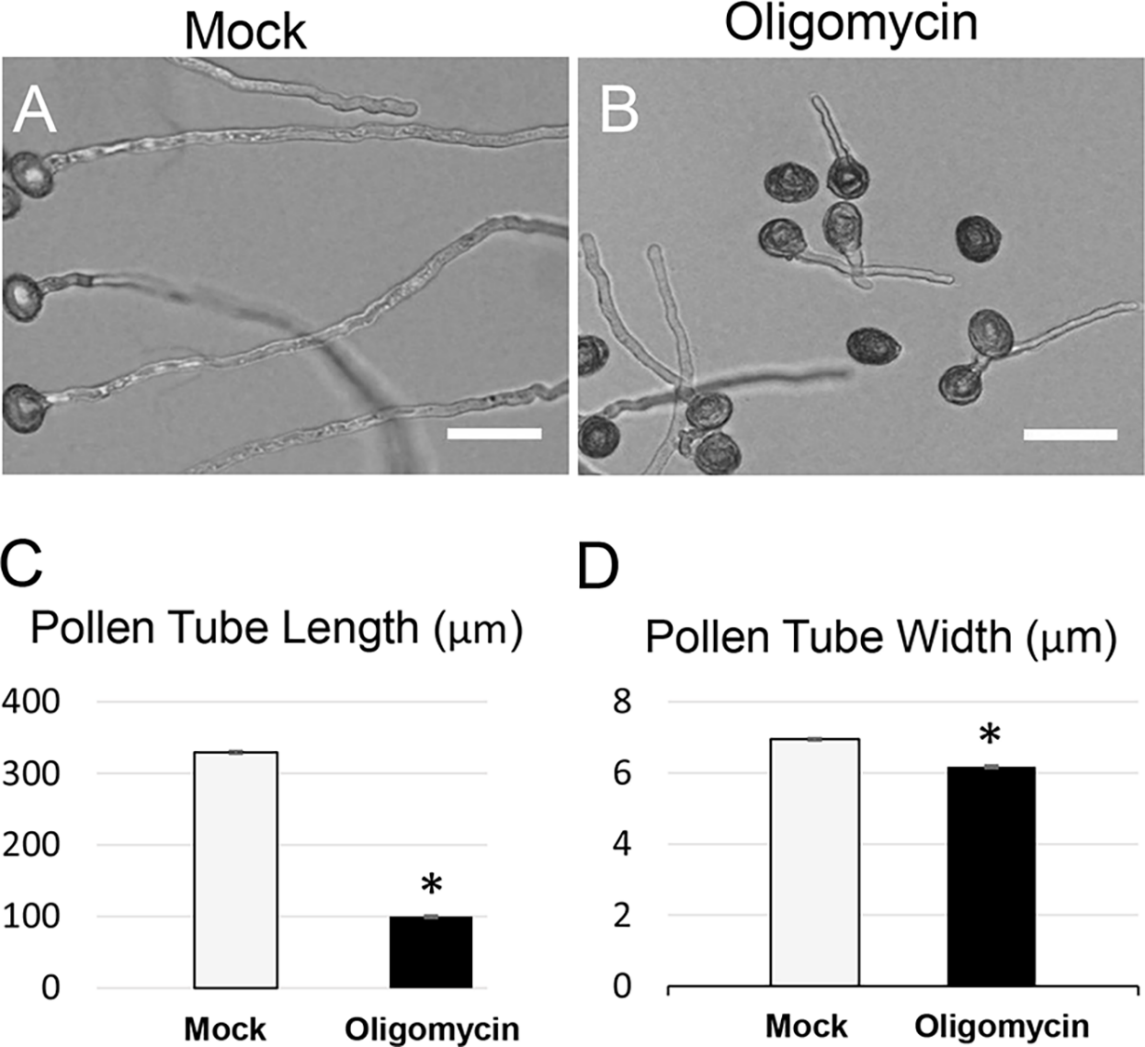
Oligomycin treatment inhibited pollen tube growth in a manner distinct from the *pgkc-1* mutant phenotype. **(A)** WT pollen tubes subjected to mock treatment. **(B)** WT pollen tubes treated with 40 nM oligomycin. Scale bar = 50μm. **(C)** Average length of 50 pollen tubes. **(D)** Average width of 50 pollen tubes. Bars represent mean ± SEM. Asterisks indicate significant differences versus mock treatment group as determined using Student’s *t*-test (* = p < 0.05).

To assess a possible relationship between cellular ATP level and pollen tube polarity, we determined whether mitochondrial respiration, the downstream pathway of glycolysis and the main source of cellular ATP production, was involved in pollen tube polarity. The potent inhibitor oligomycin has been used to block mitochondrial respiration in pollen germination medium [34]. In our assay, 40 nM oligomycin significantly inhibited WT pollen tube growth (**Fig 5A and 5B**). However, oligomycin-treated pollen tubes were uniformly short and thin, exhibiting a distinctly different phenotype than *pgkc-1* pollen tubes **(Fig 5A to 5D**). Therefore, we concluded that the *pgkc-1* pollen tube phenotype was likely not caused by inhibition of respiration.

**Fig 6:**
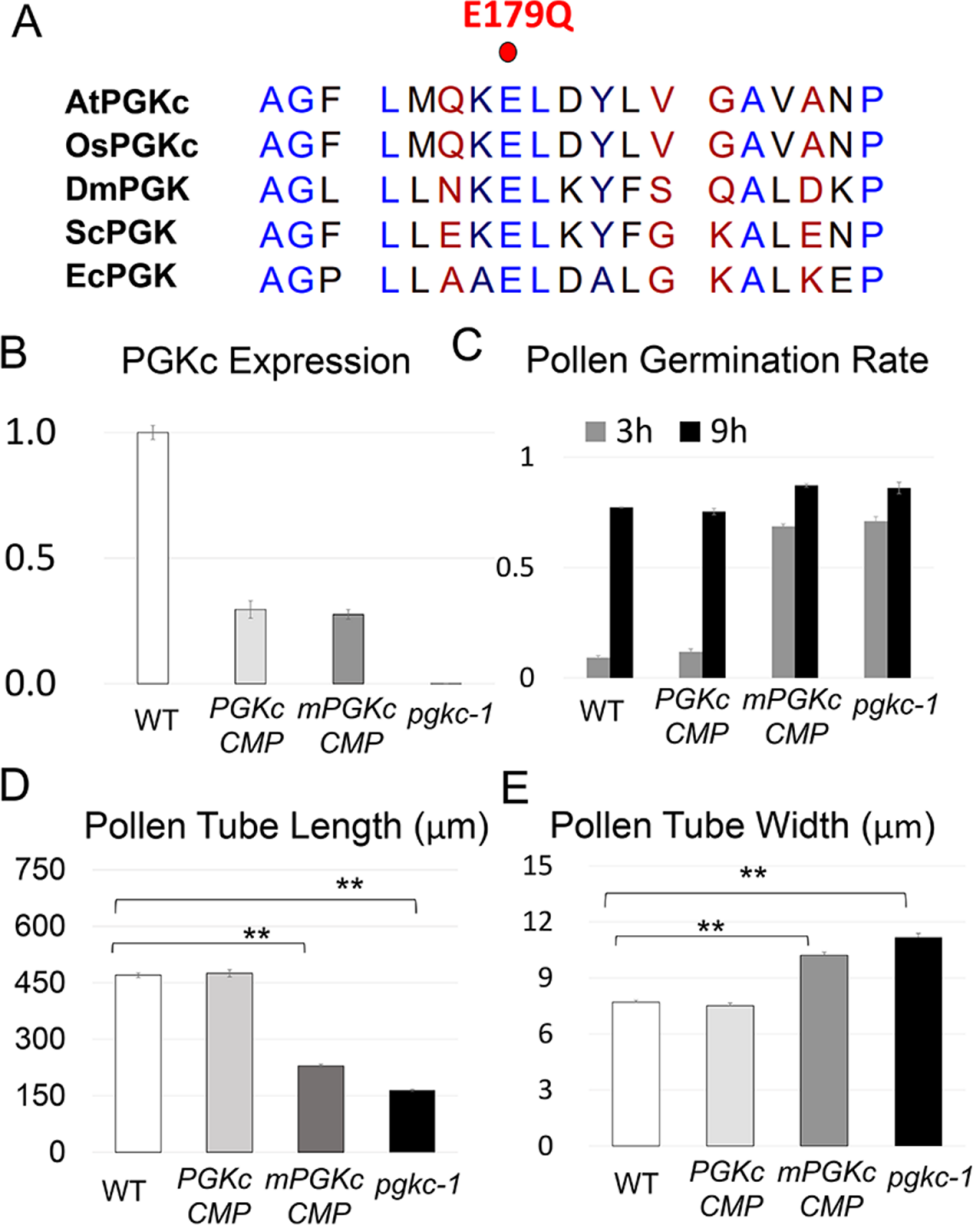
Catalytically inactive mPGKc could not rescue the *pgkc-1* mutant phenotype. **(A)** Protein sequences of the conserved Glutamate179 regions of PGK proteins. AtPGKc, *Arabidopsis thaliana* cytosolic PGK; OsPGKc, *Oryza sativa* cytosolic PGK; DmPGK, *Drosophila melanogaster* PGK; ScPGK, *Saccharomyces cerevisiae* PGK; EcPGK, *Escherichia coli* PGK. Glutamate179 in AtPGKc is labeled with a red dot. **(B)** *PGKc* expression level. **(C)** Pollen germination rate. **(D)** Average length of pollen tubes. **(E)** Average width of pollen tubes. Bars represent mean ± SEM. Asterisks indicate significant differences versus WT as determined using Student’s *t*-test (** = p < 0.001).

To check if the role of PGKc in pollen tube polarity could be attributed to its glycolytic enzymatic activity, we generated a mutant version of PGKc termed mPGKc where an evolutionally conserved residue Glutamate179 was changed to Glutamine. This mutation has been shown to impair PGK catalytic activity but not binding kinetics in yeast [35, 36] **(Fig 6A**). We introduced native promoter-driven *PGKc* or *mPGKc* cDNA into *pgkc-1* mutants, and found that WT PGKc cDNA transgene expression, while lower than native PGKc expression, was still able to complement the mutant phenotype **(Fig 6B to 6E**). In contrast, mPGKc could not rescue the mutant phenotype despite similar levels of gene expression. These results indicated that glycolytic activity was required for PGKc function in pollen tube polarity **(Fig 6B to 6E, S5 Fig**).

**Fig 7:**
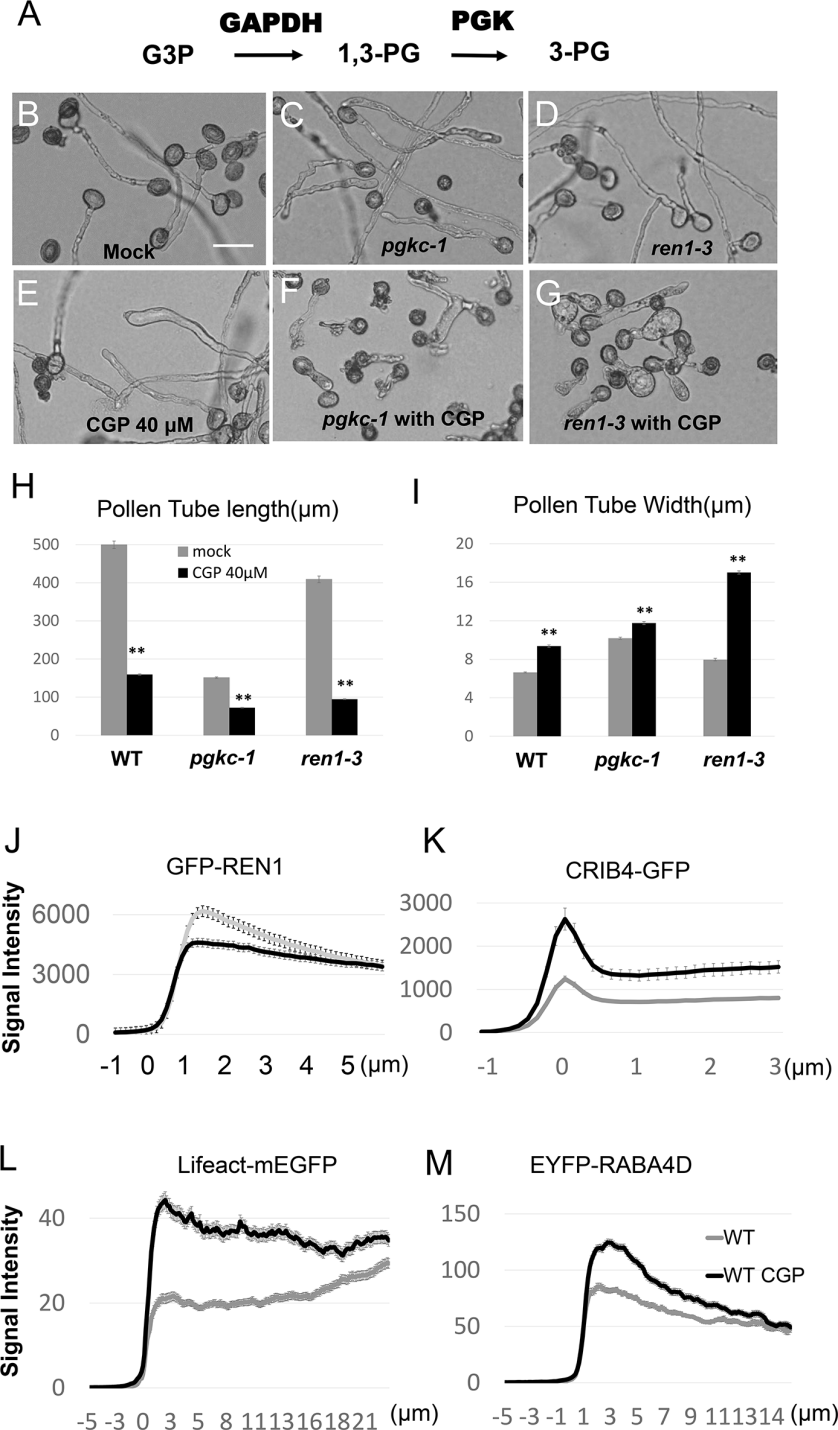
Effects of disrupting GAPDH on pollen tube polarity. **(A)** Glycolytic pathway of GAPDH and PGK. **(B to G)** Pollen tube morphology of WT **(B)**, *pgkc-1* **(C)**, and *ren1-3* **(D)** plants subjected to mock treatment; pollen tube morphology of WT **(E)**, *pgkc-1* **(F)**, and *ren1-3* **(G)** plants treated with 40 μM CGP 3466B. Both *pgkc-1* and *ren1-3* plants were dramatically depolarized by CGP medium. Scale bar = 50 μm. **(H and I)** Pollen tube length **(H)** and width **(I)** of WT, *pgkc-1*, and *ren1-3* pollen tubes subjected to mock and 40 μM CGP treatment. Bars represent mean ± SEM. Asterisks indicate significant differences versus either single mutant as determined using Student’s *t*-test with either single mutant (** = p < 0.001). **(J)-(M)** Average signal intensity along WT pollen tubes subjected to mock or CGP treatment of **(J)** GFP- REN1, **(K)** CRIB4-GFP, **(L)** Lifeact-mEGFP, **(M)** EYFP-RABA4D. Measurements were performed as described in Materials and Methods. Fifteen pollen tubes from each sample were measured. The 0 μm label indicates the position of the apical tip.

**Fig 8:**
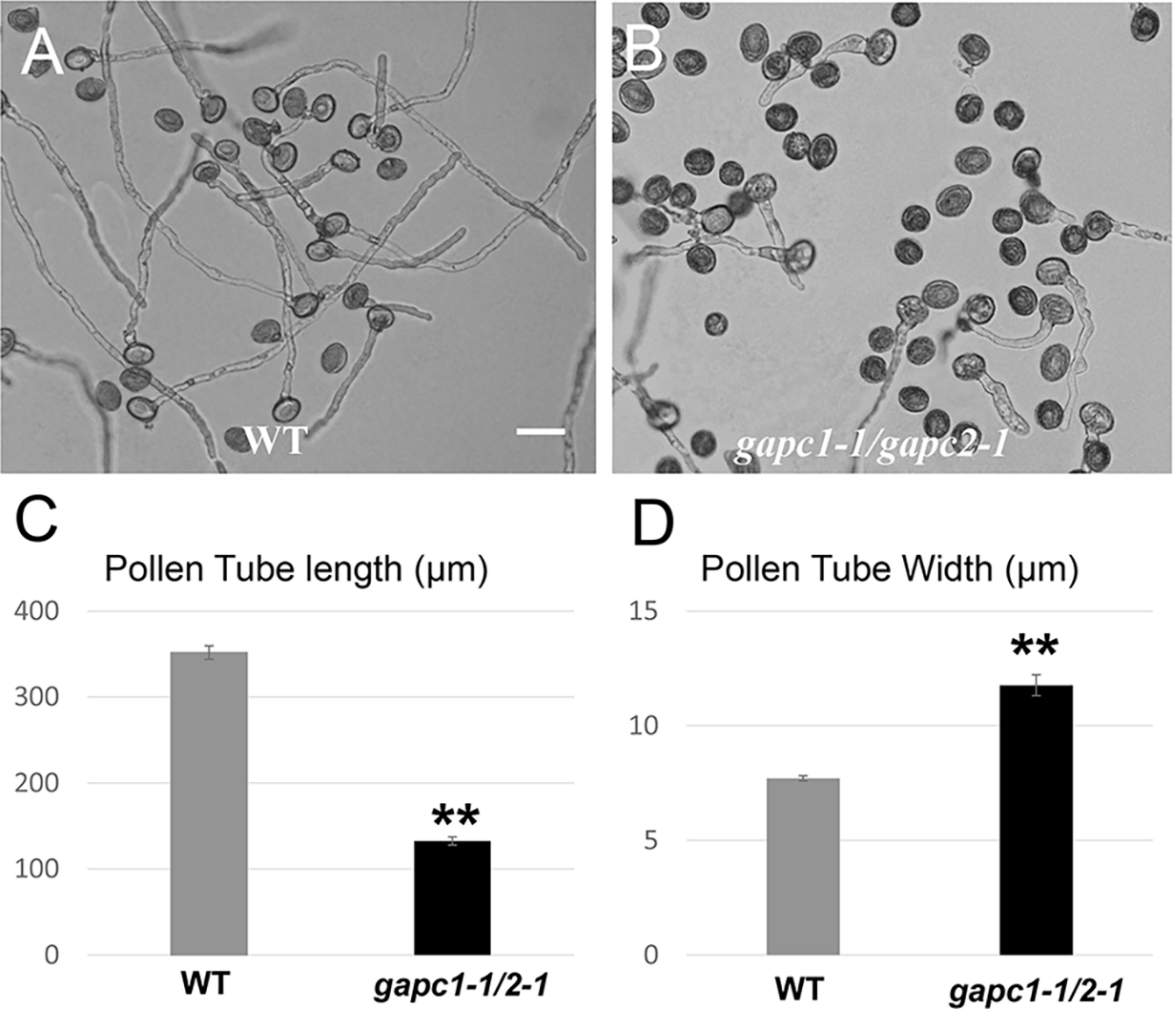
*gapcp1/gapcp2* double mutant is also defective in pollen tube polarity. **(A)** WT pollen tubes. **(B)** *gapcp1/gapcp2* double mutant pollen tubes. Scale bar = 50μm. **(C)** Average length of pollen tubes. **(D)** Average width of pollen tubes. Bars represent mean ± SEM. Asterisks indicate significant differences versus WT as determined using Student’s *t*-test (** = p < 0.001).

If PGKc regulates pollen tube polarity through its glycolytic activity, we would anticipate that other glycolytic enzymes are also involved in this process. GAPDH is an enzyme which catalyzes the conversion of glyceraldehyde-3-phosphate to 1,3BPG (**Fig 7A**). When we applied 40 μM of CGP 3466B maleate, a specific inhibitor of GAPDH [37], WT pollen tubes exhibited a *pgkc-1*-like phenotype with depolarized morphology [38](**Fig 7B, 7C and 7E**). Furthermore, when either *pgkc-1* or *ren1-3* single mutants were treated with CGP, cell polarity defect magnitude was greatly enhanced, exhibiting significantly ballooned pollen tubes (**Fig 7B to 7I**). To validate that the cellular mechanism underlying the GAPDH inhibition phenotype was similar to that underlying the *pgkc-1* mutation phenotype, we observed the distribution of GFP-REN1, CRIB4-GFP, EYFP-RABA4D and Lifeact-mEGFP in WT pollen tubes treated with 40 μM CGP 3466B. Similar to in *pgkc-1* mutants, GFP-REN1 signal was diminished while CRIB4-GFP, EYFP-RABA4D and Lifeact-mEGFP signals were enhanced in the apical region after CGP 3466B treatment (**Fig 7J to 7M**). Similarly, a double mutant of cytosolic GAPDHs, *gapc1-1/gapc2-1* [30],and the application of another GAPDH inhibitor, iodoacetate, also resulted in pollen tube phenotypes resembling that of *pgkc-1* (**Fig 8A to 8D and S6 Fig**). These results indicate that GAPDH activity is also involved in the regulation of pollen tube polarity. Taken together, we conclude that glycolysis plays an important role in the regulation of pollen tube polarity by affecting the association of the REN1 RopGAP with exocytic vesicles.

## Discussion

### Cytosolic glycolysis plays a regulatory role in pollen tube polarity

Our findings here clearly demonstrate that cytosolic glycolysis has a novel function in the regulation of cellular signaling, distinct from its conventional housekeeping role in carbon and energy metabolism. The global energy level is important for pollen development and pollen tube elongation [20–23, 31, 39]. In this study, we found that inhibition of mitochondrial respiration using oligomycin resulted in reduced pollen tube length and width. This phenotype is consistent with previous reports, while distinct from the reduced growth polarity induced by the *pgkc-1* mutation or GAPDH inhibition (**Fig 5A to 5D**).

**Fig 9:**
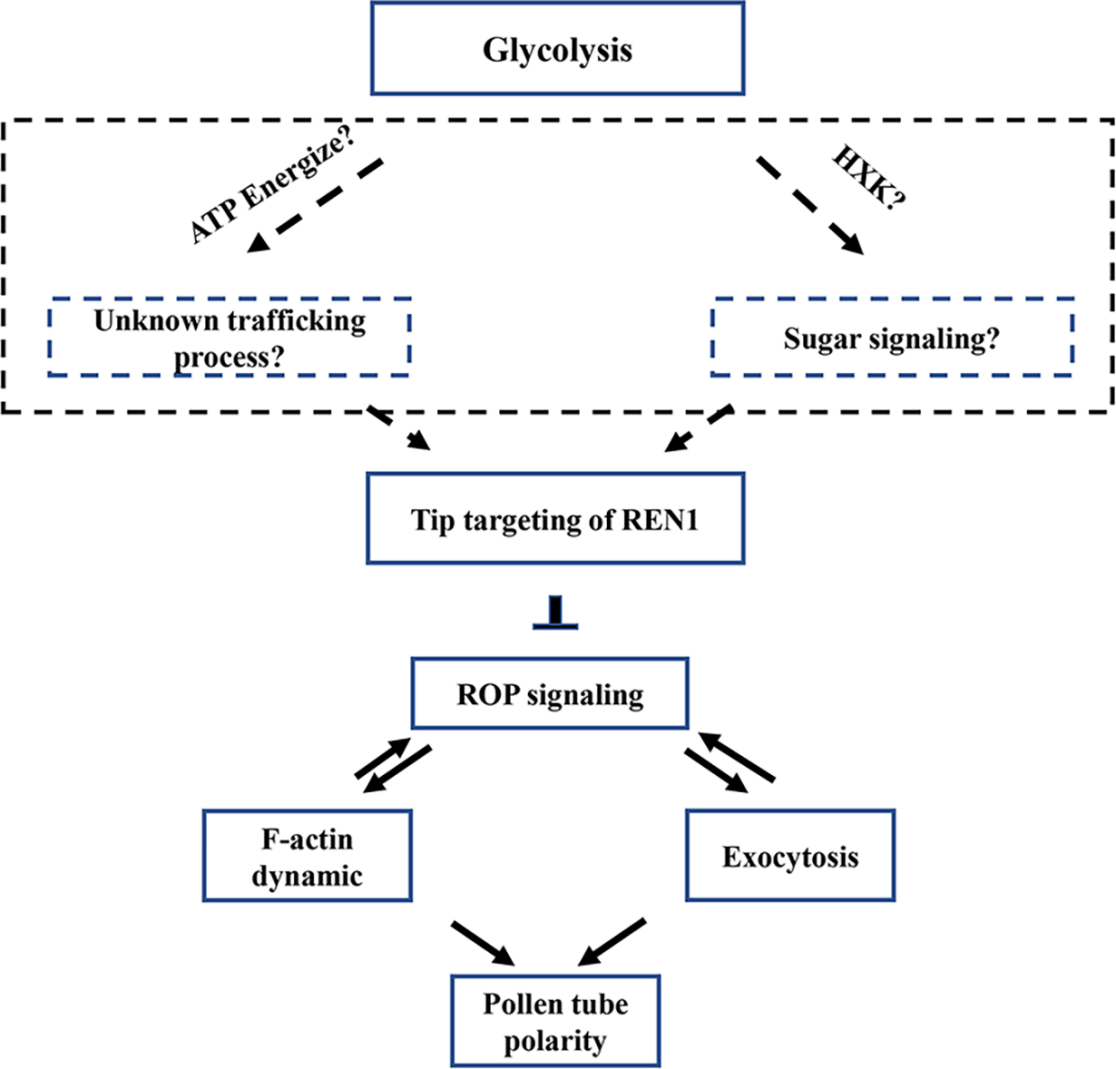
Proposed model for potential role of glycolysis in pollen tube polarity. Glycolysis is required for the association of the REN1 RopGAP with exocytic vesicles. REN1 RopGAP negatively regulates the Rho GTPase signaling, which coordinates pollen tube growth by coordinating actin dynamics and exocytosis. The mechanisms linking glycolysis with cell polarity remains elusive, which are possibly energization of unclear vesicle trafficking, or HXK signaling.

The ethanol fermentation pathway serves, concomitantly with oxidative respiration metabolism, as a bypass route to help maintain metabolic flux and energy supply in pollen tubes [25, 40]. This pathway is also downstream of glycolysis and consists of two key enzymes, pyruvate decarboxylase (PDC) and alcohol dehydrogenase (ADH) [25]. In petunia, the mutation of a pollen-specific *PDC2* gene was shown to cause reduced elongation of pollen tubes in the style, leading to a competitive disadvantage relative to WT pollen [41]. However, pollen tube polarity in *pdc2* mutants appeared to be normal [41]. Therefore, we believe that pollen tube growth polarity is modulated by a specific regulatory aspect of cytosolic glycolysis rather than glycolysis-dependent respiration or fermentation (**Fig 9**).

In animal cells, many glycolytic enzymes participate in moonlighting functions, including RNA binding, membrane fusion, cytoskeletal dynamics, autophagy, and cell death [42–46]. Similarly, cytosolic GAPDHs in plants demonstrate nuclear uracil-DNA-glycosylase activity and participate in plant immunity [47]. Here, we demonstrated that glycolytic activity is required for PGKc function in pollen tubes. Moreover, GAPDH, another enzyme in the cytosolic glycolysis pathway, plays a similar role as PGKc in pollen tube polarity. Based on these results, it is more likely that it is the glycolysis pathway which regulates pollen tube polarity, rather than a moonlighting function of a glycolytic enzyme (**Fig 9**).

### Cytosolic glycolysis regulates Rho GTPase signaling and downstream cellular activities

The pollen tube polarity defects present in the *pgkc* mutant have been associated with the over-activation of ROP1, as well as the over-accumulation of F-actin and exocytic vesicles in the tip region. Previous findings have suggested that REN1-based negative feedback globally inhibited ROP1, as ROP1 activity is dependent upon the association of REN1 with exocytic vesicles at the apical plasma membrane [14]. Both *ren1* mutation and constitutively active ROP1 (CA-ROP1) expression has been shown to cause ROP1 hyper-activation, leading to F-actin stabilization, apical cortex vesicle accumulation, and pollen tube depolarization [11, 14]. In the *pgkc* mutant or during treatment with a GAPDH inhibitor, the association between REN1 and the exocytic vesicles is abolished, thus accounting for the observed over-activation of ROP1. Accordingly, tip region over-accumulation of F-actin and exocytic vesicles appears to be attributed to ROP activation (**Fig 9**).

The oscillation of apical ROP1 activity is regulated by positive and negative feedback via F-actin-mediated exocytosis [7]. Could the aberrant REN1 localization be the consequence of disrupted F-actin in the *pgkc-1* mutant, rather than the cause? According to previous studies, if the loss of PGKc activity simply enhance F-actin accumulation, then one may expect overall alteration of pollen tube elongation, rather than polarity. Mutations of F-actin severing factors RIC1 or MAP18, also caused aberrant F-actin overaccumulation in the apical tip of pollen tubes [27, 48]. However, *ric1* mutant exhibited enhanced elongation and *map18* is defective in growth direction of pollen tubes, while the pollen tube polarity was normal in both cases [27, 48]. Therefore, we interpret disrupted RhoGTPase signaling in the *pgkc* pollen tubes as a reason rather than consequence of the aberrant cellular activities (**Fig 9**). Nevertheless, our study does not exclude the possibility that *pgkc* mutation might directly interrupt other unelucidated cellular processes, which simultaneously affect multiple steps in the feedback loops of RhoGTPase signaling, including REN1 distribution, F-actin dynamics, and exocytic vesicle trafficking.

### Possible mechanisms linking glycolysis with cell polarity

Several possible underlying mechanisms may link the glycolysis pathway with pollen tube polarity. Mitochondria provide most of the energy required by the cell. However, mitochondria are not evenly distributed in polarized cells, and may not meet the needs of all organelles [49, 50]. In contrast, although net energy gain is low, glycolysis could produce ATP close to energy sinks, thus complementing mitochondrial function. For instance, in neurons, the vesicles in fast axonal transport are energized by on-board ATP provided by specifically localized glycolytic machinery rather than mitochondrial respiration [51]. Mitochondria are absent from the apical tip of pollen tubes, where PGKc is present [50, 52]. Therefore, it is possible that cytosolic glycolysis may provide an ATP source in close proximity to some unclear vesicle activities, similar to the fast axonal transport, which are required for the targeting and/or trafficking of REN1 protein in pollen tube tips (**Fig 9**).

Glycolysis is a fundamental energy metabolism pathway, but glycolytic enzymes and intermediates may also play important signaling roles in growth and development. One of the most important signaling hubs is the enzyme hexokinase (HXK). As the first enzyme in glycolysis, HXK is able to phosphorylate glucose, producing glucose-6-phosphate [53]. Independent of its catalytic activity, plant HXK has also been proven as a glucose sensor for the regulation of sugar metabolism and signaling pathways [54, 55]. Since PGK and GAPDH are downstream of HXK and aldolase, there is a possibility that PGKc or GAPDH inhibition might cause accumulation of glucose in pollen tube, resulting in a hyperactivation of HXK signaling in pollen tubes (**Fig 9**). It would be helpful to examine this possibility in the future by overexpressing HXK in pollen tubes.

It is less likely but still possible that glycolysis may regulate pollen tube polarity through signaling by downstream intermediate metabolites, such as 3-phosphoglyceric acid (3PG), a product of PGKs. However, given that the plastidial glycolysis pathway remains intact in *pgkc* mutant pollen tubes, metabolic intermediates are unlikely to be deficient. Consistent with this, adding 3PG or pyruvate did not affect the pollen tube polarity phenotype of the *pgkc* mutant, even at concentrations inhibitory to WT pollen tubes (**S7 Fig and S8 Fig**). Although we could not clarify whether exogenous metabolites could substitute for intracellular metabolic intermediates under our experimental conditions, this result indicates that these metabolites have no effect on pollen tube polarity. Nonetheless, future studies are needed to elucidate the mechanisms by which cytosolic glycolysis regulates the association of REN1 with apical vesicles and subsequent cell polarity modulation in pollen tubes.

## Materials and methods

### Plant materials and mutant screening

*Arabidopsis* (Columbia ecotype) were used as WT specimens. All plants were grown under a 16 h photoperiod at 22°C. SALK collections of individually indexed homozygous T-DNA insertion lines were obtained from the ABRC (http://signal.salk.edu/cgi-bin/homozygotes.cgi). For *in vitro* pollen germination screening, 5–10 seeds from each SALK line were grown in individual pots. Pollen grains from three plants for each line were collected and germinated in *in vitro* germination medium as previously described [14, 56]. Lines with the pollen tube polarity phenotype were selected as mutant candidates for further verification. SALK_066422C (*pgkc-1*) was identified during screening. Another allele, SALK_062377 (*pgkc-2*), was obtained from the ABRC. Genotyping was performed based on the protocol provided on the SALK website. *gapc1-1/gapc2-1* double mutant seeds were gifts from Dr. Xueming Wang, and genotype was confirmed using primers as described [30]. All primers used are listed in Supplemental S1 Table

### RT-PCR

Total RNA was extracted from indicated tissues using the E.Z.N.A. RNA extraction kit (Omega) according to manufacturer’s instructions. Oligo dT-primed cDNA was synthesized from 500 mg of total RNA using the PrimeScript RT reagent Kit with gDNA Eraser (Takara). Quantitative PCR analysis was performed with the SYBR Premix Ex Taq II ROX plus kit (Takara) using a Mx3005 device (Agilent). Relative levels of each transcript were calculated after being normalized to UBC21 endogenous control.

### Plasmid construction

All constructs were generated using Gateway technology (Invitrogen). Primers used are listed in Supplemental S1 Table. All entry vectors were generated from the pDONR-zeo vector (Invitrogen). LR reactions were conducted using LR Clonase II (Invitrogen) with corresponding entry vectors and destination vectors.

To construct catalytic inactive *mPGKc* complementation vector, *PGKc* cDNA was cloned first. Then DpnI-mediated site-directed mutagenesis was performed to change the G535 to C [35, 57]. pGWB604 vector was modified by inserting a 2.1 kb *PGKc* promoter with HindIII and SbfI. LR cloning were then performed to generate the *proPGKc*::*PGKc-GFP* or *proPGKc*::*mPGKc-GFP* constructs, respectively.

### Observation of *Arabidopsis* pollen germination and pollen tube growth

Open flowers were collected and pollen grains were dusted onto standard agar-germination medium with 18% sucrose, 0.01% boric acid, 1 mM CaCl_2_, 1 mM Ca(NO_3_)_2_, 1 mM MgSO_4_, pH 6.0, and 0.5% Difc Noble agar (BD Biosciences). Incubation times ranged from 2 to 9 h at 23°C, and pollen tubes were observed under an Imager M2 inverted microscope (Olympus). Tube length and width were measured using ImageJ software. Since the morphology of *pgkc-1* pollen tubes was non-uniform, pollen tube width was measured at the widest point.50-100 pollen grains or pollen tubes were measured.

For pollen tube chemical treatment, LatB (Invitrogen), BFA(Invitrogen), iodoacetate (Sigma), oligomycin (Sigma), CGP 3466B (Tocris Bioscience) at indicated concentrations were added to the above solid pollen germination medium. WT and mutant pollen grains were germinated using the same medium and compared side-by-side.

For Lifeact-mEGFP, EYFP-RABA4D, REN1-GFP marker observation, previously reported marker lines were used for crossing with *pgkc-1* mutants. Double homozygote F2 progeny were identified by genotyping for the presence of mutant *pgkc-1* and observation of pollen GFP/YFP fluorescent signal, respectively. For the CRIB4-GFP marker, as *pgkc-1* and CRIB4-GFP are linked on the same chromosome, a vector containing CRIB4-GFP was used to transform *pgkc-1*. The chosen line was then backcrossed with WT, and CRIB4-GFP on a WT background was obtained as part of F2 progeny. Fluorescent microscopy was performed with a Spinning Disk Confocal Microscope Andor Revolution WD.

To quantitatively measure GFP signal intensity, the ImageJ line profile tool was used according to user guidelines. Briefly, a five pixel block was drawn from the background toward the tip along the axis of a pollen tube. The signal intensity along the line was measured by the line profile tool. The apical tip was defined as the position where signal intensity was two-fold greater than the black background, and this position was designated as 0 μm. Fifteen to twenty pollen tubes were measured for each sample, with the data from each pollen tube aligned by tip position, and average intensities were calculated.

## Supporting information

S1 Fig *pgkc* allele mutants exhibit pollen tube polarity phenotype. **(A)** Diagram of *PGKc* gene structure and T-DNA insertion sites. **(B)** Genotyping result to validate of *pgkc-1* and *pgkc*-2 homozygous plants. **(C)** Q-RT-PCR of PGKc gene expression in *pgkc-1* and *pgkc*-2 homozygous plants. **(D)** Q-RT-PCR of *PGKc* gene expression in complemented plants. Pollen tube phenotype of **(E)** WT and **(F)** *pgkc-1* progeny genotype plant in F2 progenies, and **(G)** *pgkc*-2 mutant. Scale bar = 50μm.
(TIF)

S2 Fig Plant Morphology of *pgkc-1* and WT mutant. **(A)** Flowering plants at 40 days after sawing. **(B)** Plants at 21 days after sawing. **(C)** Flower morphology.
(TIF)

S3 Fig Subcellular localization of PGK-GFP fusion proteins in Arabidopsis transgenic plants. **(A)** Free GFP, **(B)** AT1G79550 (PGKc), **(C)** AT3G12780, **(D)** AT1G56190 in leaves of transgenic plants. **(E)** Free GFP and **(F)** PGKc-GFP in pollen tube. Protein fusions were conducted by fusing GFP at C terminus of proteins, which are driven by 35S promoter in **(B)**-**(D)**, and Lat52 promoter in **(F)**, Scale bars = 10μm.
(TIF)

S4 Fig Genotyping and expression analysis of *ren1-3* mutant. **(A)** Diagram of *REN1* gene structure and T-DNA insertion sites. Bold arrow indicates T-DNA insertion site of *ren1-3* mutant. Grey slim arrows indicate primers for RT-PCR upstream of insertion site. Black slim arrows indicate primers for RT-PCR spanning the insertion site. **(B)** Genotyping result to validate of *ren1-3* homozygous plants. **(C)** Expression of *REN1* fragment upstream of T-DNA insertion in seedlings. **(D)** Expression of *REN1* fragment spanning T-DNA insertion in seedlings.
(TIF)

S5 Fig Catalytically inactive mPGKc could not rescue the *pgkc-1* mutant phenotype. **(A)** WT pollen tube morphology. **(B)** *pgkc-1* pollen tube morphology. **(C)** Complemented *pgkc-1* pollen tube. **(D)** Complementation with *mPGKc*.
(TIF)

S6 Fig Effects of disrupting GAPDH on pollen tube polarity. **(A)** WT pollen tubes on mock medium. **(B)** WT pollen tubes on 1μM iodoacetate, an inhibitor of GAPDH. Scale bar = 50μm.
(TIF)

S7 Fig Exogenous 3PG could not affect pollen tube polarity. **(A)** WT and *pgkc-1* pollen tube morphology on different concentration of 3PG. Scale bar = 50μm. **(B)** Quantitative data of pollen tube length. **(C)** Quantitative data of pollen tube width. Bars represent means +/- SEM. Asterisks indicates significant differences in comparison with mock (** p<0.001). Students T test.
(TIF)

S8 Fig Exogenous pyruvates could not affect pollen tube polarity. **(A)** WT and *pgkc-1* pollen tube morphology on different concentration of pyruvate. Scale bar = 50μm. **(B)** Quantitative data of pollen tube length. **(C)** Quantitative data of pollen tube width. Bars represent means +/**-** SEM. Asterisks indicates significant differences in comparison with mock (** p<0.001). Students T test.
(TIF)

S1 Table Primers used in this study. (XLSX)

S2 Table Raw numerical data statistic analysis. (XLSX)

## References

[1] Muller M, Mentel M, van Hellemond JJ, Henze K, Woehle C, Gould SB, et al Biochemistry and evolution of anaerobic energy metabolism in eukaryotes. Microbiol Mol Biol Rev. 2012;76(2):444–95. doi: 10.1128/MMBR.05024-11 ; PubMed Central PMCID: PMCPMC3372258. 22688819 10.1128/MMBR.05024-11 PMC3372258

[2] Vidali L, Rounds CM, Hepler PK, Bezanilla M. Lifeact-mEGFP reveals a dynamic apical F-actin network in tip growing plant cells. PLoS One. 2009;4(5):e5744 doi: 10.1371/journal.pone.0005744 ; PubMed Central PMCID: PMC2684639. 19478943 10.1371/journal.pone.0005744 PMC2684639

[3] Brenman JE. AMPK/LKB1 signaling in epithelial cell polarity and cell division. Cell Cycle. 2007;6(22):2755–9. doi: 10.4161/cc.6.22.4927 . 17986859 10.4161/cc.6.22.4927

[4] Hardie DG. AMP-activated protein kinase: an energy sensor that regulates all aspects of cell function. Genes Dev. 2011;25(18):1895–908. doi: 10.1101/gad.17420111 ; PubMed Central PMCID: PMCPMC3185962. 21937710 10.1101/gad.17420111 PMC3185962

[5] Zhang CS, Hawley SA, Zong Y, Li M, Wang Z, Gray A, et al Fructose-1,6-bisphosphate and aldolase mediate glucose sensing by AMPK. Nature. 2017;548(7665):112–6. doi: 10.1038/nature23275 ; PubMed Central PMCID: PMCPMC5544942. 28723898 10.1038/nature23275 PMC5544942

[6] Mirouse V, Billaud M. The LKB1/AMPK polarity pathway. FEBS Lett. 2011;585(7):981–5. doi: 10.1016/j.febslet.2010.12.025 . 21185289 10.1016/j.febslet.2010.12.025

[7] Guan Y, Guo J, Li H, Yang Z. Signaling in pollen tube growth: crosstalk, feedback, and missing links. Mol Plant. 2013;6(4):1053–64. doi: 10.1093/mp/sst070 ; PubMed Central PMCID: PMC3842152. 23873928 10.1093/mp/sst070 PMC3842152

[8] Hepler PK, Vidali L, Cheung AY. Polarized cell growth in higher plants. Annual review of cell and developmental biology. 2001;17:159–87. doi: 10.1146/annurev.cellbio.17.1.159 . 11687487 10.1146/annurev.cellbio.17.1.159

[9] Gibbon BC, Kovar DR, Staiger CJ. Latrunculin B has different effects on pollen germination and tube growth. Plant Cell. 1999;11(12):2349–63. ; PubMed Central PMCID: PMC144132. 10590163 10.1105/tpc.11.12.2349 PMC144132

[10] Wang H, Zhuang X, Cai Y, Cheung AY, Jiang L. Apical F-actin-regulated exocytic targeting of NtPPME1 is essential for construction and rigidity of the pollen tube cell wall. Plant J. 2013;76(3):367–79. doi: 10.1111/tpj.12300 . 23906068 10.1111/tpj.12300

[11] Lee YJ, Szumlanski A, Nielsen E, Yang Z. Rho-GTPase-dependent filamentous actin dynamics coordinate vesicle targeting and exocytosis during tip growth. J Cell Biol. 2008;181(7):1155–68. doi: 10.1083/jcb.200801086 ; PubMed Central PMCID: PMCPMC2442199. 18591430 10.1083/jcb.200801086 PMC2442199

[12] Zhang Y, McCormick S. The regulation of vesicle trafficking by small GTPases and phospholipids during pollen tube growth. Sex Plant Reprod. 2010;23(2):87–93. doi: 10.1007/s00497-009-0118-z . 20490965 10.1007/s00497-009-0118-z

[13] Qin Y, Yang Z. Rapid tip growth: insights from pollen tubes. Semin Cell Dev Biol. 2011;22(8):816–24. doi: 10.1016/j.semcdb.2011.06.004 ; PubMed Central PMCID: PMCPMC3210868. 21729760 10.1016/j.semcdb.2011.06.004 PMC3210868

[14] Hwang JU, Vernoud V, Szumlanski A, Nielsen E, Yang Z. A tip-localized RhoGAP controls cell polarity by globally inhibiting Rho GTPase at the cell apex. Current biology: CB. 2008;18(24):1907–16. doi: 10.1016/j.cub.2008.11.057 ; PubMed Central PMCID: PMC2615002. 19108776 10.1016/j.cub.2008.11.057 PMC2615002

[15] Lin Y, Yang Z. Inhibition of pollen tube elongation by microinjected anti-Rop1Ps antibodies suggests a crucial role for Rho-Type GTPases in the control of tip growth. Plant Cell. 1997;9(9):1647–59. doi: 10.1105/tpc.9.9.1647 ; PubMed Central PMCID: PMCPMC157040. 12237397 10.1105/tpc.9.9.1647 PMC157040

[16] Li H, Lin Y, Heath RM, Zhu MX, Yang Z. Control of pollen tube tip growth by a Rop GTPase-dependent pathway that leads to tip-localized calcium influx. Plant Cell. 1999;11(9):1731–42. ; PubMed Central PMCID: PMCPMC144310. 10488239 10.1105/tpc.11.9.1731 PMC144310

[17] Zhang Y, McCormick S. A distinct mechanism regulating a pollen-specific guanine nucleotide exchange factor for the small GTPase Rop in *Arabidopsis thaliana*. Proc Natl Acad Sci U S A. 2007;104(47):18830–5. doi: 10.1073/pnas.0705874104 ; PubMed Central PMCID: PMCPMC2141862. 18000057 10.1073/pnas.0705874104 PMC2141862

[18] Zhang Y, McCormick S. Regulation of pollen tube polarity: Feedback loops rule. Plant Signal Behav. 2008;3(5):345–7. ; PubMed Central PMCID: PMCPMC2634279. 19841667 10.4161/psb.3.5.5353 PMC2634279

[19] Feng QN, Kang H, Song SJ, Ge FR, Zhang YL, Li E, et al Arabidopsis RhoGDIs are critical for cellular homeostasis of pollen tubes. Plant Physiol. 2016;170(2):841–56. doi: 10.1104/pp.15.01600 ; PubMed Central PMCID: PMCPMC4734571. 26662604 10.1104/pp.15.01600 PMC4734571

[20] Selinski J, Konig N, Wellmeyer B, Hanke GT, Linke V, Neuhaus HE, et al The plastid-localized NAD-dependent malate dehydrogenase is crucial for energy homeostasis in developing Arabidopsis thaliana seeds. Mol Plant. 2014;7(1):170–86. doi: 10.1093/mp/sst151 . 24198233 10.1093/mp/sst151

[21] Zhao Z, Assmann SM. The glycolytic enzyme, phosphoglycerate mutase, has critical roles in stomatal movement, vegetative growth, and pollen production in Arabidopsis thaliana. J Exp Bot. 2011;62(14):5179–89. doi: 10.1093/jxb/err223 ; PubMed Central PMCID: PMCPMC3193020. 21813794 10.1093/jxb/err223 PMC3193020

[22] Munoz-Bertomeu J, Cascales-Minana B, Irles-Segura A, Mateu I, Nunes-Nesi A, Fernie AR, et al The plastidial glyceraldehyde-3-phosphate dehydrogenase is critical for viable pollen development in Arabidopsis. Plant Physiol. 2010;152(4):1830–41. doi: 10.1104/pp.109.150458 ; PubMed Central PMCID: PMCPMC2850016. 20107025 10.1104/pp.109.150458 PMC2850016

[23] Prabhakar V, Lottgert T, Geimer S, Dormann P, Kruger S, Vijayakumar V, et al Phosphoenolpyruvate provision to plastids is essential for gametophyte and sporophyte development in Arabidopsis thaliana. Plant Cell. 2010;22(8):2594–617. doi: 10.1105/tpc.109.073171 ; PubMed Central PMCID: PMCPMC2947176. 20798327 10.1105/tpc.109.073171 PMC2947176

[24] Dickinson DB. Germination of Lily pollen: respiration and tube growth. Science. 1965;150(3705):1818–9. doi: 10.1126/science.150.3705.1818 . 17841976 10.1126/science.150.3705.1818

[25] Rounds CM, Winship LJ, Hepler PK. Pollen tube energetics: respiration, fermentation and the race to the ovule. AoB PLANTS. 2011;2011:plr019 doi: 10.1093/aobpla/plr019 ; PubMed Central PMCID: PMC3169925. 22476489 10.1093/aobpla/plr019 PMC3169925

[26] Rosa-Tellez S, Anoman AD, Flores-Tornero M, Toujani W, Alseek S, Fernie AR, et al Phosphoglycerate kinases are co-regulated to adjust metabolism and to optimize growth. Plant Physiol. 2018;176(2):1182–98. doi: 10.1104/pp.17.01227 ; PubMed Central PMCID: PMCPMC5813584. 28951489 10.1104/pp.17.01227 PMC5813584

[27] Zhou Z, Shi H, Chen B, Zhang R, Huang S, Fu Y. Arabidopsis RIC1 severs actin filaments at the apex to regulate pollen tube growth. Plant Cell. 2015;27(4):1140–61. doi: 10.1105/tpc.114.135400 ; PubMed Central PMCID: PMC4558691. 25804540 10.1105/tpc.114.135400 PMC4558691

[28] de Graaf BH, Cheung AY, Andreyeva T, Levasseur K, Kieliszewski M, Wu HM. Rab11 GTPase-regulated membrane trafficking is crucial for tip-focused pollen tube growth in tobacco. Plant Cell. 2005;17(9):2564–79. doi: 10.1105/tpc.105.033183 ; PubMed Central PMCID: PMCPMC1197435. 16100336 10.1105/tpc.105.033183 PMC1197435

[29] Szumlanski AL, Nielsen E. The Rab GTPase RabA4d regulates pollen tube tip growth in Arabidopsis thaliana. Plant Cell. 2009;21(2):526–44. doi: 10.1105/tpc.108.060277 ; PubMed Central PMCID: PMC2660625. 19208902 10.1105/tpc.108.060277 PMC2660625

[30] Guo L, Ma F, Wei F, Fanella B, Allen DK, Wang X. Cytosolic phosphorylating glyceraldehyde-3-phosphate dehydrogenases affect Arabidopsis cellular metabolism and promote seed oil accumulation. Plant Cell. 2014;26(7):3023–35. doi: 10.1105/tpc.114.126946 ; PubMed Central PMCID: PMC4145129. 24989043 10.1105/tpc.114.126946 PMC4145129

[31] Selinski J, Scheibe R. Pollen tube growth: where does the energy come from? Plant Signal Behav. 2014;9(12):e977200 doi: 10.4161/15592324.2014.977200 ; PubMed Central PMCID: PMC4622831. 25482752 10.4161/15592324.2014.977200 PMC4622831

[32] Wang Q, Kong L, Hao H, Wang X, Lin J, Samaj J, et al Effects of brefeldin A on pollen germination and tube growth. Antagonistic effects on endocytosis and secretion. Plant Physiol. 2005;139(4):1692–703. doi: 10.1104/pp.105.069765 ; PubMed Central PMCID: PMCPMC1310552. 16299176 10.1104/pp.105.069765 PMC1310552

[33] Rutten TL, Knuiman B. Brefeldin A effects on tobacco pollen tubes. European journal of cell biology. 1993;61(2):247–55. . 8223715

[34] Chang DT, Reynolds IJ. Mitochondrial trafficking and morphology in healthy and injured neurons. Prog Neurobiol. 2006;80(5):241–68. doi: 10.1016/j.pneurobio.2006.09.003 . 17188795 10.1016/j.pneurobio.2006.09.003

[35] Mas MT, Resplandor ZE, Riggs AD. Site-directed mutagenesis of glutamate-190 in the hinge region of yeast 3-phosphoglycerate kinase: implications for the mechanism of domain movement. Biochemistry. 1987;26(17):5369–77. . 2890374 10.1021/bi00391a023

[36] Wilson CA, Hardman N, Fothergill-Gilmore LA, Gamblin SJ, Watson HC. Yeast phosphoglycerate kinase: investigation of catalytic function by site-directed mutagenesis. Biochem J. 1987;241(2):609–14. ; PubMed Central PMCID: PMCPMC1147603. 3297036 10.1042/bj2410609 PMC1147603

[37] Kragten E, Lalande I, Zimmermann K, Roggo S, Schindler P, Muller D, et al Glyceraldehyde-3-phosphate dehydrogenase, the putative target of the antiapoptotic compounds CGP 3466 and R-(-)-deprenyl. J Biol Chem. 1998;273(10):5821–8. . 9488718 10.1074/jbc.273.10.5821

[38] Williamson JR. Glycolytic control mechanisms. 3. Effects of iodoacetamide and fluoroacetate on glucose metabolism in the perfused rat heart. J Biol Chem. 1967;242(19):4476–85. . 4229046

[39] Rounds CM, Hepler PK, Fuller SJ, Winship LJ. Oscillatory growth in lily pollen tubes does not require aerobic energy metabolism. Plant Physiol. 2010;152(2):736–46. doi: 10.1104/pp.109.150896 ; PubMed Central PMCID: PMC2815890. 20007440 10.1104/pp.109.150896 PMC2815890

[40] Tadege M, Kuhlemeier C. Aerobic fermentation during tobacco pollen development. Plant Mol Biol. 1997;35(3):343–54. . 9349258 10.1023/a:1005837112653

[41] Gass N, Glagotskaia T, Mellema S, Stuurman J, Barone M, Mandel T, et al Pyruvate decarboxylase provides growing pollen tubes with a competitive advantage in petunia. Plant Cell. 2005;17(8):2355–68. doi: 10.1105/tpc.105.033290 ; PubMed Central PMCID: PMC1182494. 15994907 10.1105/tpc.105.033290 PMC1182494

[42] Jeffery CJ. Moonlighting proteins. Trends Biochem Sci. 1999;24(1):8–11. . 10087914 10.1016/s0968-0004(98)01335-8

[43] Kumagai H, Sakai H. A porcine brain protein (35 K protein) which bundles microtubules and its identification as glyceraldehyde 3-phosphate dehydrogenase. J Biochem. 1983;93(5):1259–69. . 6885722 10.1093/oxfordjournals.jbchem.a134260

[44] Tisdale EJ. Glyceraldehyde-3-phosphate dehydrogenase is required for vesicular transport in the early secretory pathway. J Biol Chem. 2001;276(4):2480–6. doi: 10.1074/jbc.M007567200 . 11035021 10.1074/jbc.M007567200

[45] Singh R, Green MR. Sequence-specific binding of transfer RNA by glyceraldehyde-3-phosphate dehydrogenase. Science. 1993;259(5093):365–8. . 8420004 10.1126/science.8420004

[46] Meyer-Siegler K, Mauro DJ, Seal G, Wurzer J, deRiel JK, Sirover MA. A human nuclear uracil DNA glycosylase is the 37-kDa subunit of glyceraldehyde-3-phosphate dehydrogenase. Proc Natl Acad Sci U S A. 1991;88(19):8460–4. ; PubMed Central PMCID: PMCPMC52528. 1924305 10.1073/pnas.88.19.8460 PMC52528

[47] Henry E, Fung N, Liu J, Drakakaki G, Coaker G. Beyond glycolysis: GAPDHs are multi-functional enzymes involved in regulation of ROS, autophagy, and plant immune responses. PLoS Genet. 2015;11(4):e1005199 doi: 10.1371/journal.pgen.1005199 ; PubMed Central PMCID: PMC4412566. 25918875 10.1371/journal.pgen.1005199 PMC4412566

[48] Zhu L, Zhang Y, Kang E, Xu Q, Wang M, Rui Y, et al MAP18 regulates the direction of pollen tube growth in Arabidopsis by modulating F-actin organization. Plant Cell. 2013;25(3):851–67. doi: 10.1105/tpc.113.110528 ; PubMed Central PMCID: PMCPMC3634693. 23463774 10.1105/tpc.113.110528 PMC3634693

[49] MacAskill AF, Kittler JT. Control of mitochondrial transport and localization in neurons. Trends Cell Biol. 2010;20(2):102–12. doi: 10.1016/j.tcb.2009.11.002 . 20006503 10.1016/j.tcb.2009.11.002

[50] Hepler PK, Winship LJ. The pollen tube clear zone: clues to the mechanism of polarized growth. J Integr Plant Biol. 2015;57(1):79–92. doi: 10.1111/jipb.12315 . 25431342 10.1111/jipb.12315

[51] Zala D, Hinckelmann MV, Yu H, Lyra da Cunha MM, Liot G, Cordelieres FP, et al Vesicular glycolysis provides on-board energy for fast axonal transport. Cell. 2013;152(3):479–91. doi: 10.1016/j.cell.2012.12.029 . 23374344 10.1016/j.cell.2012.12.029

[52] Colaco R, Moreno N, Feijo JA. On the fast lane: mitochondria structure, dynamics and function in growing pollen tubes. J Microsc. 2012;247(1):106–18. doi: 10.1111/j.1365-2818.2012.03628.x . 22681536 10.1111/j.1365-2818.2012.03628.x

[53] Claeyssen E, Rivoal J. Isozymes of plant hexokinase: occurrence, properties and functions. Phytochemistry. 2007;68(6):709–31. doi: 10.1016/j.phytochem.2006.12.001 . 17234224 10.1016/j.phytochem.2006.12.001

[54] Moore B, Zhou L, Rolland F, Hall Q, Cheng WH, Liu YX, et al Role of the Arabidopsis glucose sensor HXK1 in nutrient, light, and hormonal signaling. Science. 2003;300(5617):332–6. doi: 10.1126/science.1080585 . 12690200 10.1126/science.1080585

[55] Sheen J. Master regulators in plant glucose signaling networks. J Plant Biol. 2014;57(2):67–79. doi: 10.1007/s12374-014-0902-7 ; PubMed Central PMCID: PMCPMC4270195. 25530701 10.1007/s12374-014-0902-7 PMC4270195

[56] Guan Y, Meng X, Khanna R, LaMontagne E, Liu Y, Zhang S. Phosphorylation of a WRKY transcription factor by MAPKs is required for pollen development and function in Arabidopsis. PLoS Genet. 2014;10(5):e1004384 doi: 10.1371/journal.pgen.1004384 ; PubMed Central PMCID: PMCPMC4022456. 24830428 10.1371/journal.pgen.1004384 PMC4022456

[57] Fisher CL, Pei GK. Modification of a PCR-based site-directed mutagenesis method. Biotechniques. 1997;23(4):570–1, 4. . 9343663 10.2144/97234bm01

