# Supplementary figures and images for: Glycolysis regulates pollen tube polarity via Rho GTPase signaling
Source: PLoS Genet. 2018 Apr 27;14(4):e1007373. doi: 10.1371/journal.pgen.1007373 (PMC5942846; doi:10.1371/journal.pgen.1007373)

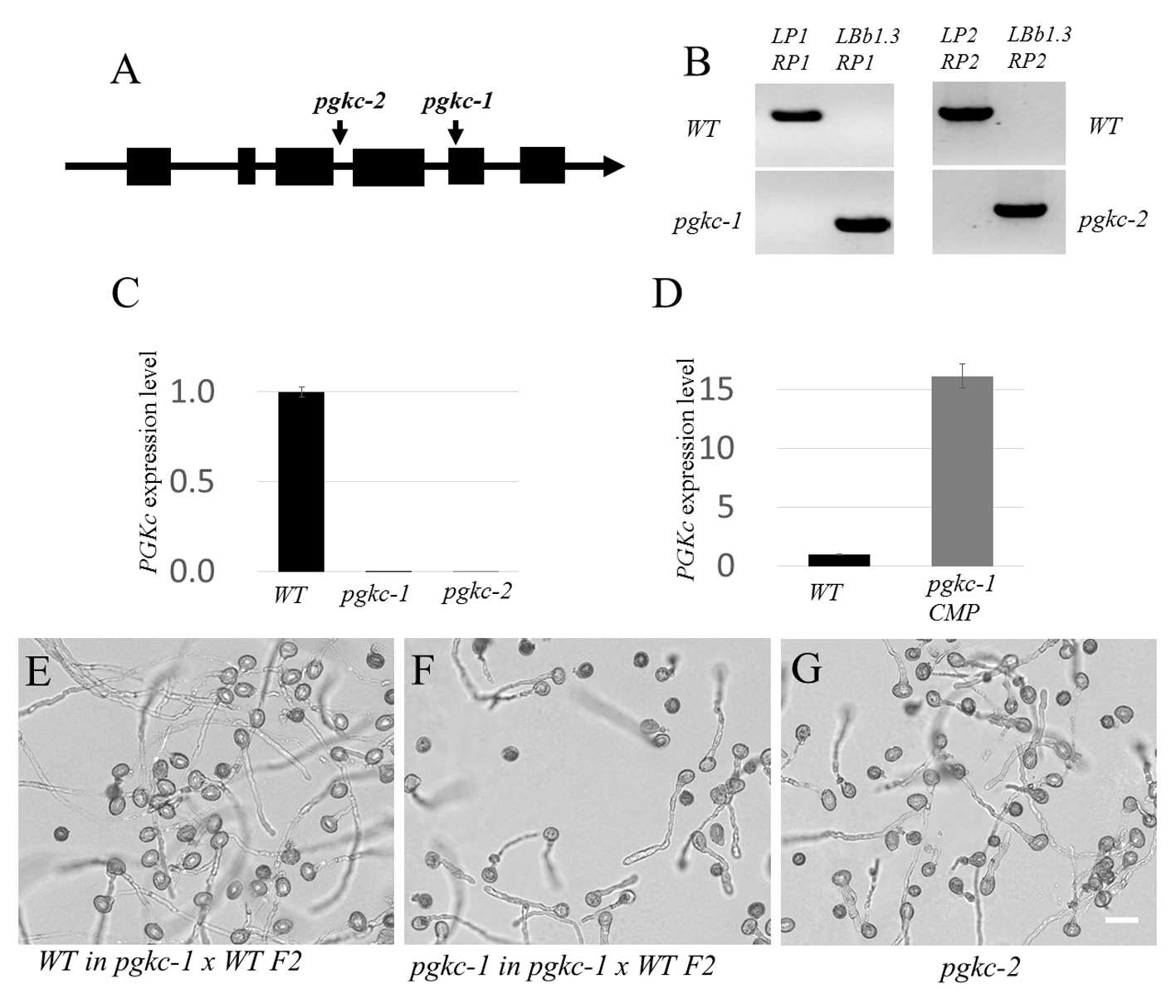

Supplement: S1 Fig — (A) Diagram of PGKc gene structure and T-DNA insertion sites. (B) Genotyping result to validate of pgkc-1 and pgkc-2 homozygous plants. (C) Q-RT-PCR of PGKc gene expression in pgkc-1 and pgkc-2 homozygous plants. (D) Q-RT-PCR of PGKc gene expression in complemented plants. Pollen tube phenotype of (E) WT and (F) pgkc-1 progeny genotype plant in F2 progenies, and (G) pgkc-2 mutant. Scale bar = 50μm. (TIF) [file pgen.1007373.s001.tif]

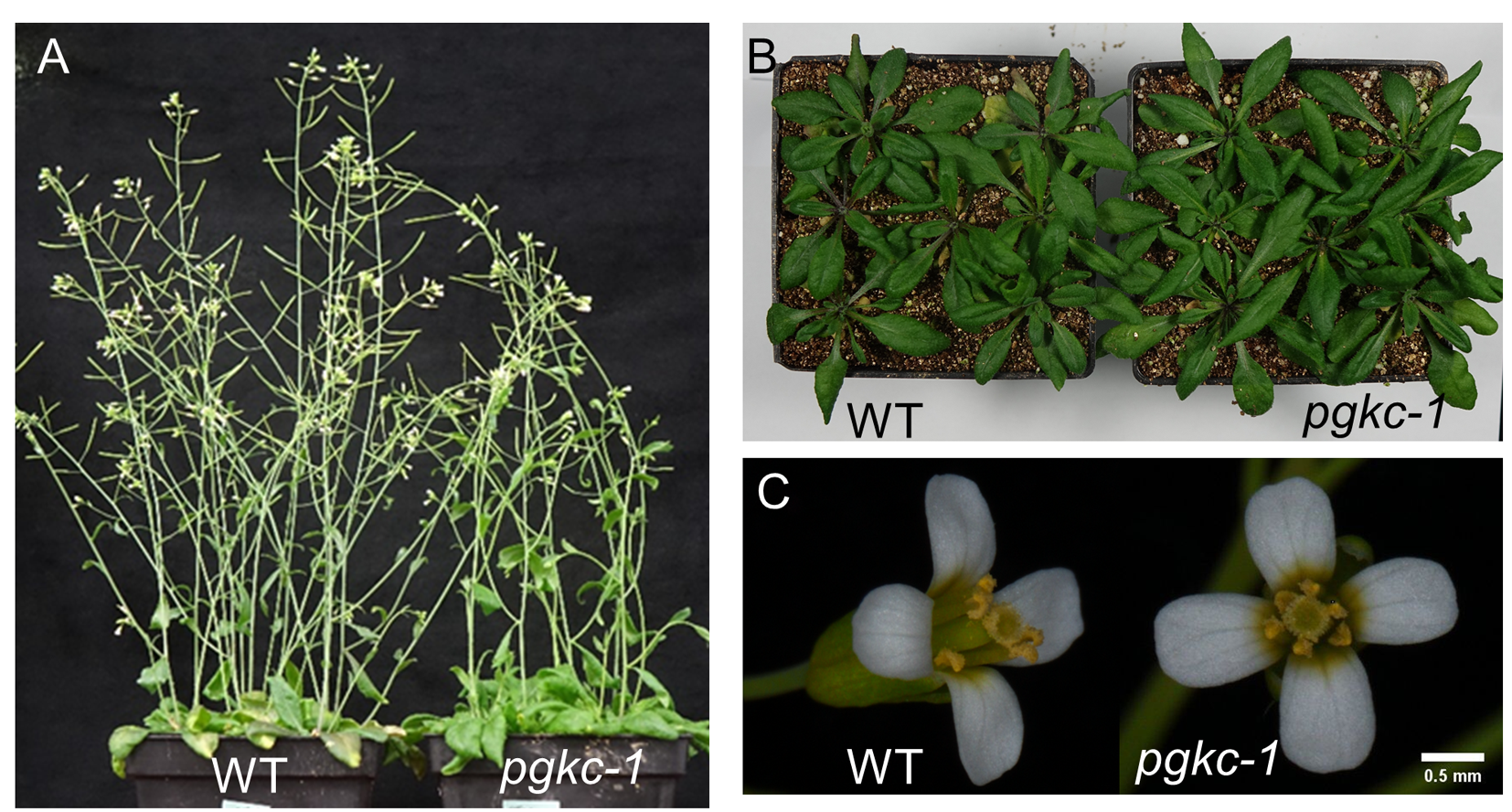

Supplement: S2 Fig — (A) Flowering plants at 40 days after sawing. (B) Plants at 21 days after sawing. (C) Flower morphology. (TIF) [file pgen.1007373.s002.tif]

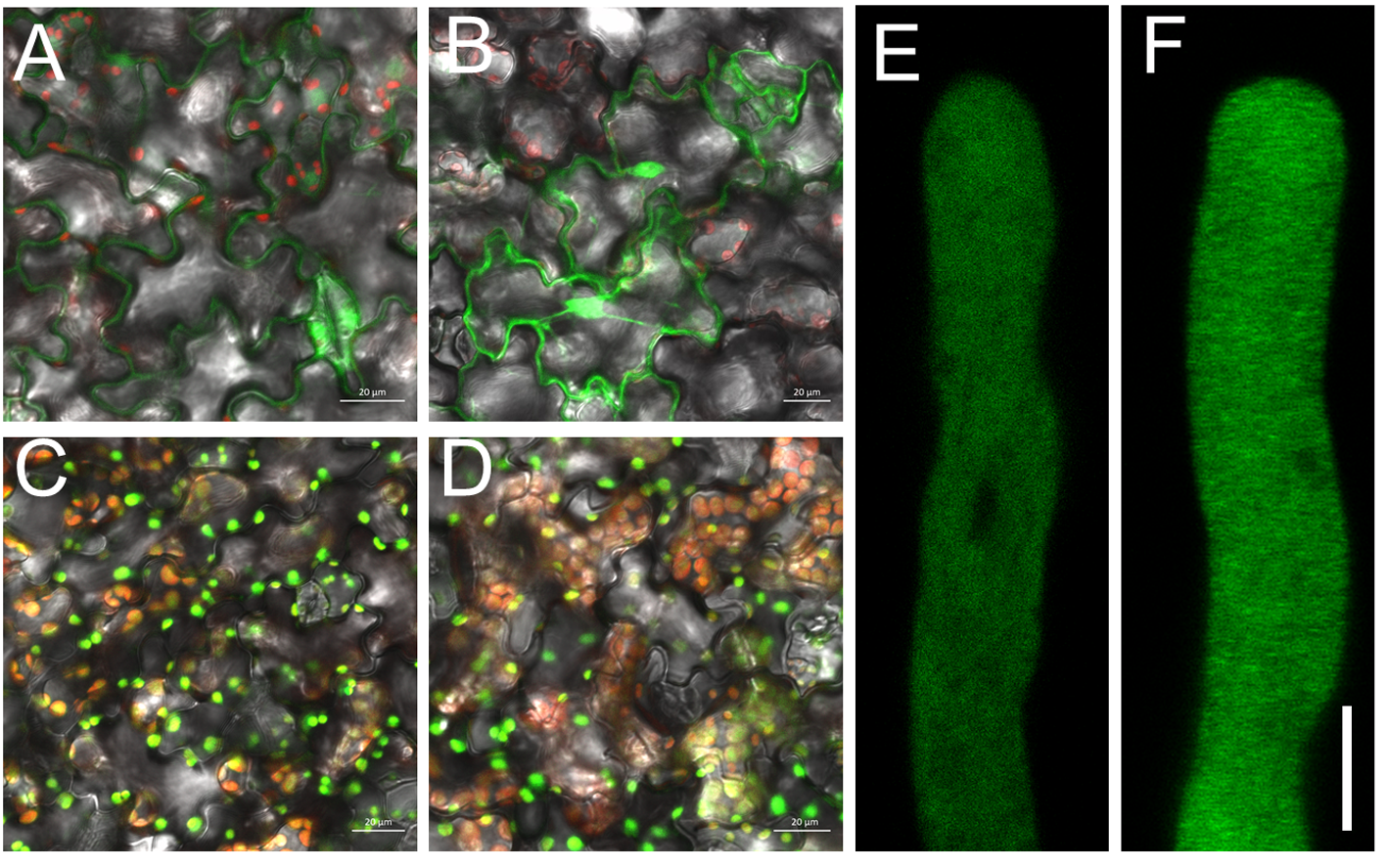

Supplement: S3 Fig — (A) Free GFP, (B) AT1G79550 (PGKc), (C) AT3G12780, (D) AT1G56190 in leaves of transgenic plants. (E) Free GFP and (F) PGKc-GFP in pollen tube. Protein fusions were conducted by fusing GFP at C terminus of proteins, which are driven by 35S promoter in (B)-(D), and Lat52 promoter in (F), Scale bars = 10μm. (TIF) [file pgen.1007373.s003.tif]

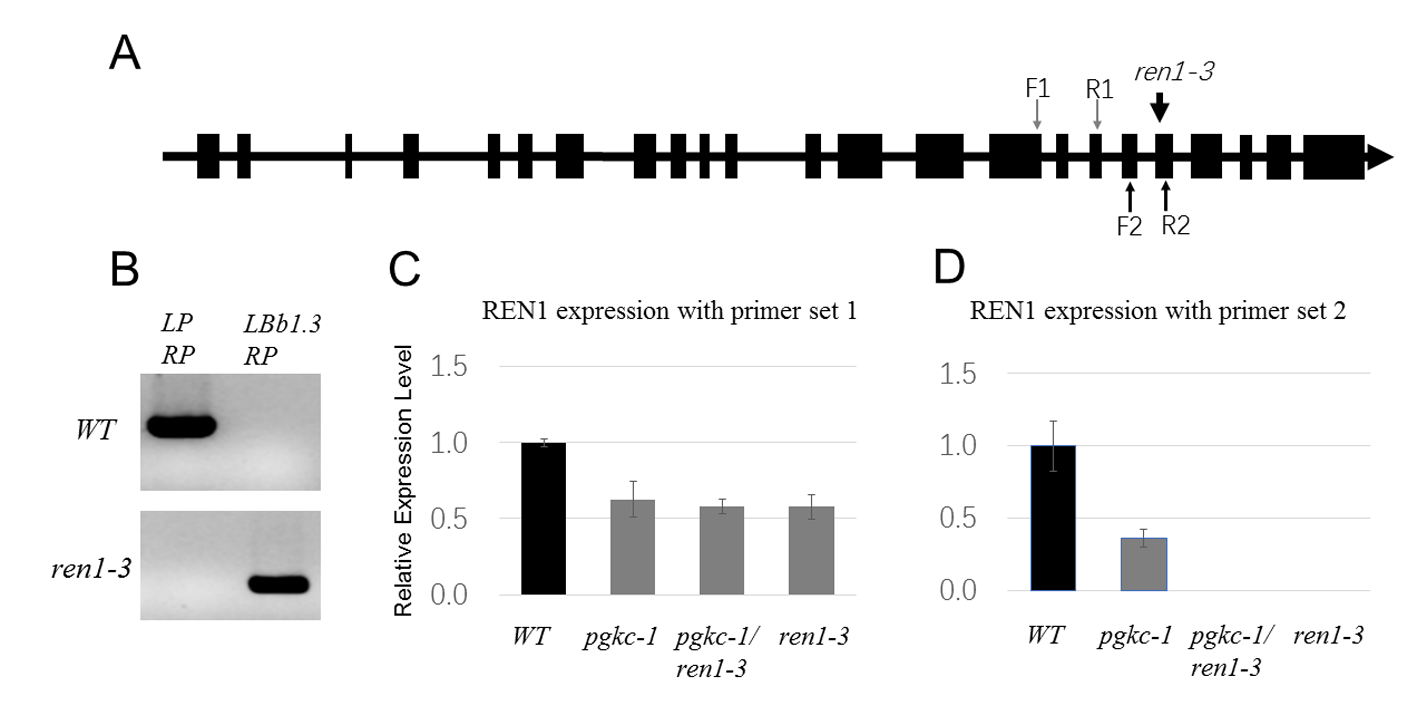

Supplement: S4 Fig — (A) Diagram of REN1 gene structure and T-DNA insertion sites. Bold arrow indicates T-DNA insertion site of ren1-3 mutant. Grey slim arrows indicate primers for RT-PCR upstream of insertion site. Black slim arrows indicate primers for RT-PCR spanning the insertion site. (B) Genotyping result to validate of ren1-3 homozygous plants. (C) Expression of REN1 fragment upstream of T-DNA insertion in seedlings. (D) Expression of REN1 fragment spanning T-DNA insertion in seedlings. (TIF) [file pgen.1007373.s004.tif]

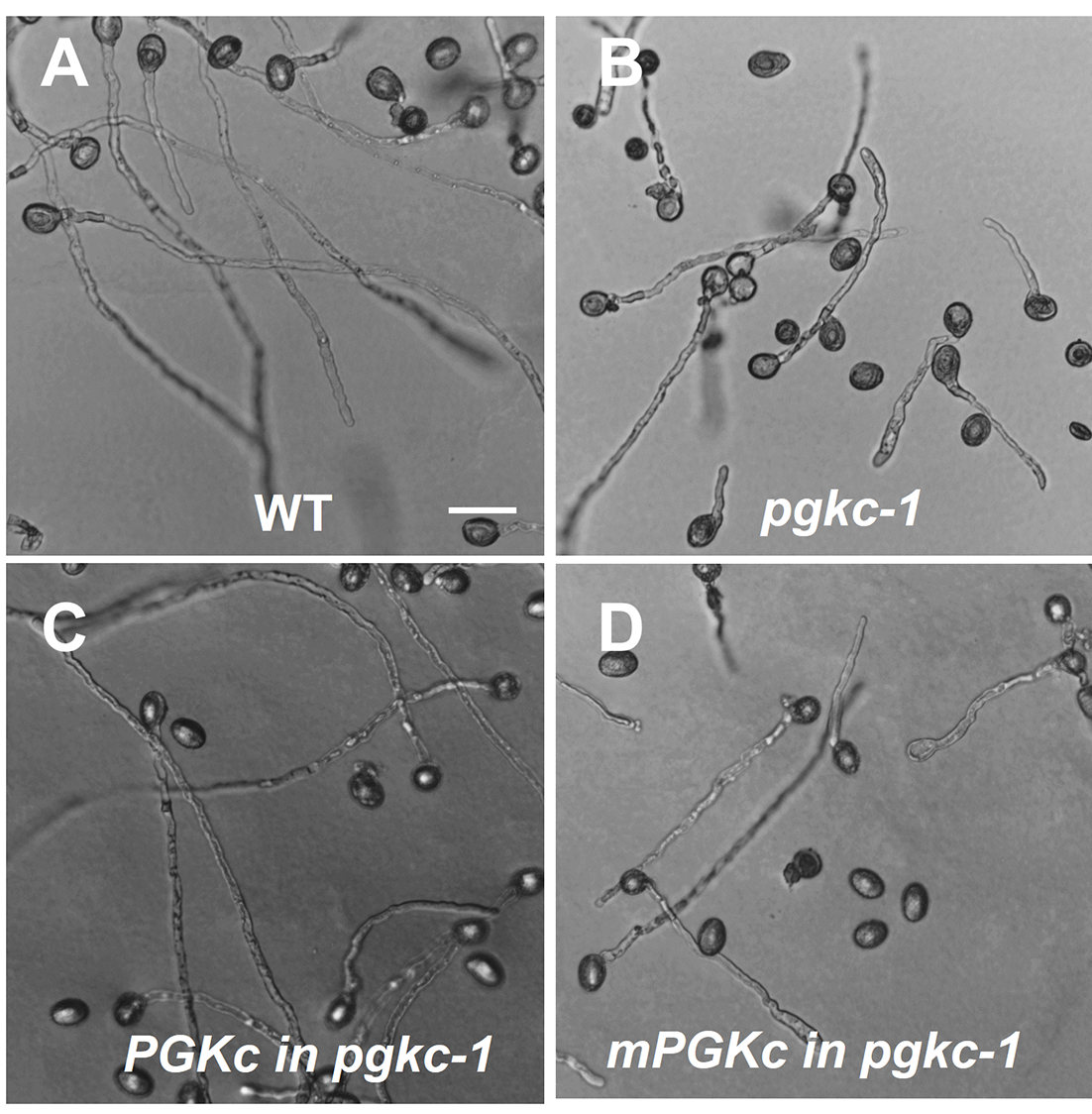

Supplement: S5 Fig — (A) WT pollen tube morphology. (B) pgkc-1 pollen tube morphology. (C) Complemented pgkc-1 pollen tube. (D) Complementation with mPGKc. (TIF) [file pgen.1007373.s005.tif]

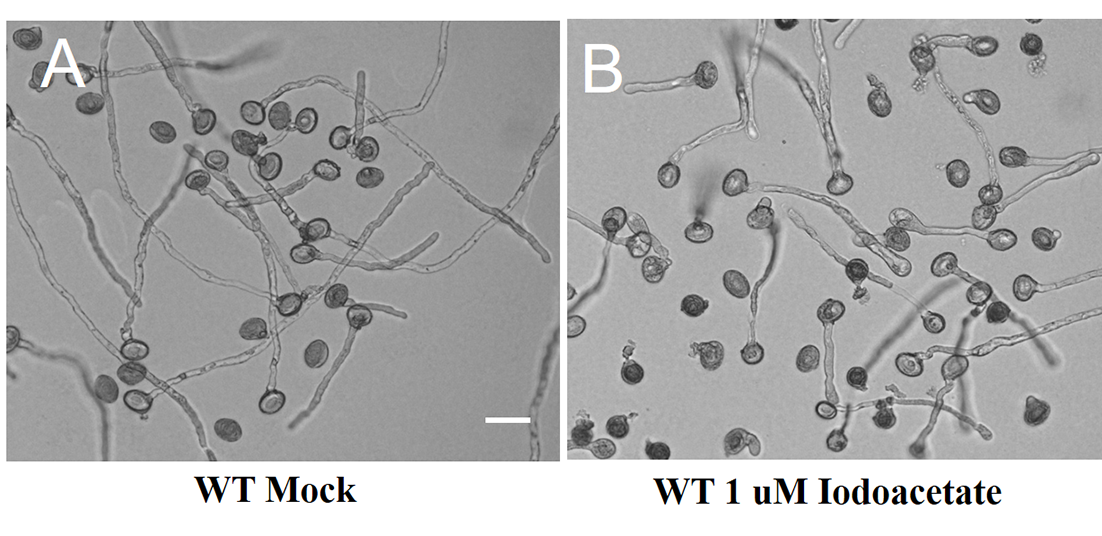

Supplement: S6 Fig — (A) WT pollen tubes on mock medium. (B) WT pollen tubes on 1μM iodoacetate, an inhibitor of GAPDH. Scale bar = 50μm. (TIF) [file pgen.1007373.s006.tif]

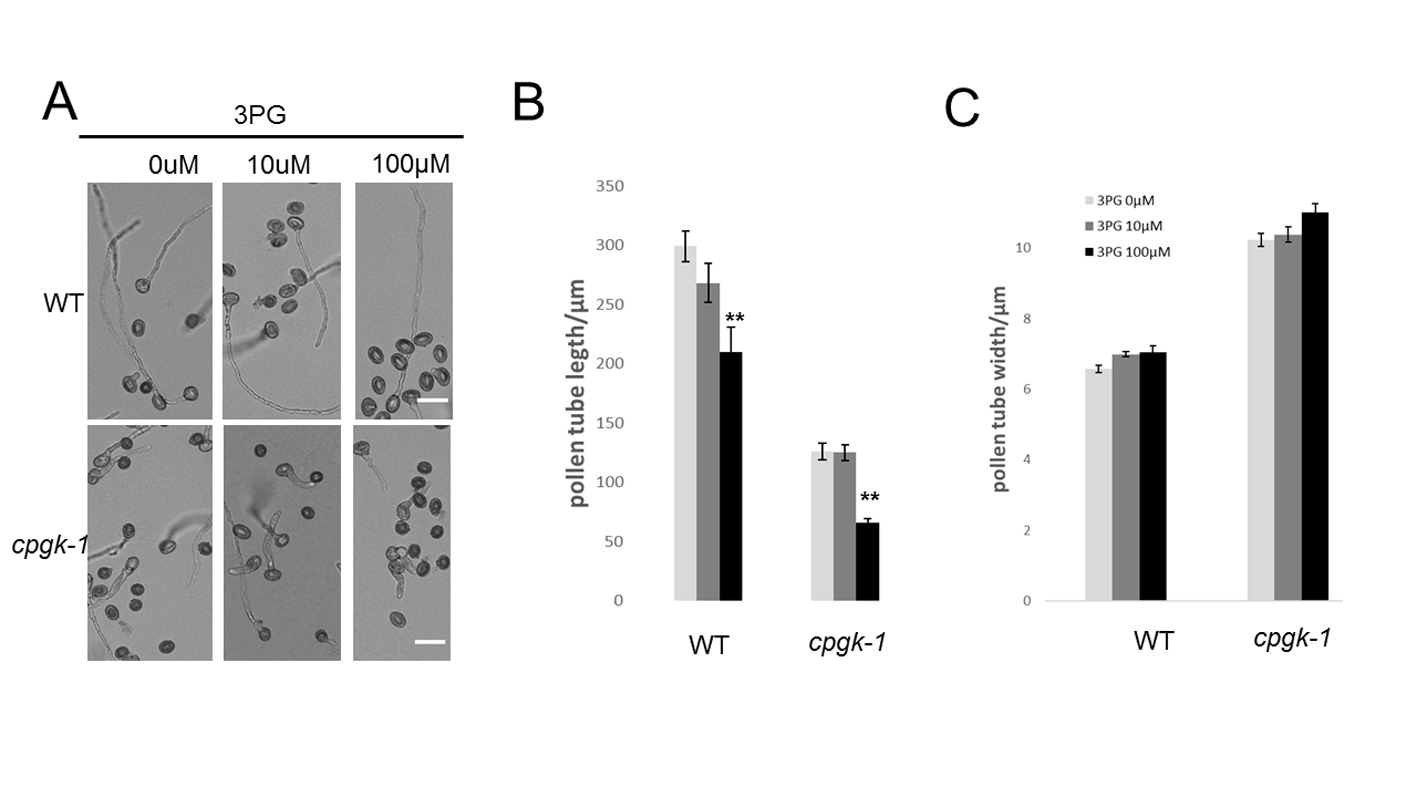

Supplement: S7 Fig — (A) WT and pgkc-1 pollen tube morphology on different concentration of 3PG. Scale bar = 50μm. (B) Quantitative data of pollen tube length. (C) Quantitative data of pollen tube width. Bars represent means +/- SEM. Asterisks indicates significant differences in comparison with mock (** p<0.001). Students T test. (TIF) [file pgen.1007373.s007.tif]

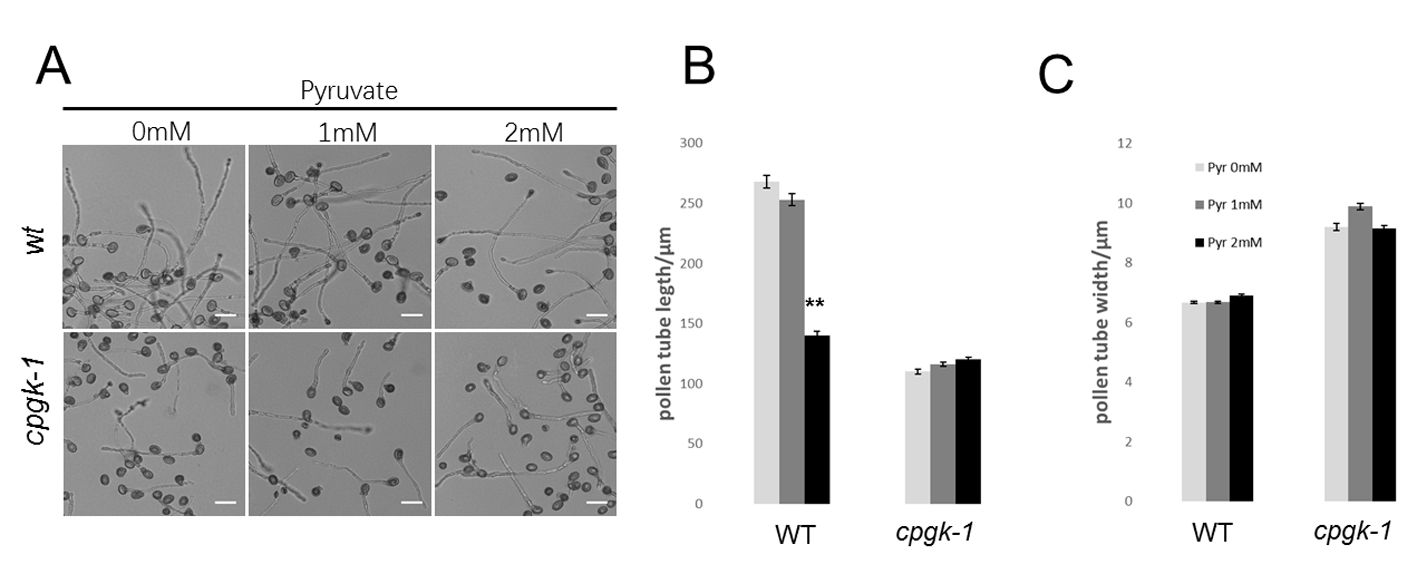

Supplement: S8 Fig — (A) WT and pgkc-1 pollen tube morphology on different concentration of pyruvate. Scale bar = 50μm. (B) Quantitative data of pollen tube length. (C) Quantitative data of pollen tube width. Bars represent means +/- SEM. Asterisks indicates significant differences in comparison with mock (** p<0.001). Students T test. (TIF) [file pgen.1007373.s008.tif]
